# Attracting Dynamics of Frontal Cortex Ensembles during Memory-Guided Decision-Making

**DOI:** 10.1371/journal.pcbi.1002057

**Published:** 2011-05-19

**Authors:** Emili Balaguer-Ballester, Christopher C. Lapish, Jeremy K. Seamans, Daniel Durstewitz

**Affiliations:** 1 Bernstein-Center for Computational Neuroscience Heidelberg-Mannheim, Central Institute of Mental Health, Medical Faculty Mannheim, Heidelberg University, Mannheim, Germany; 2 Department of Psychology, Indiana University-Purdue University, Indianapolis, Indiana, United States of America; 3 Brain Research Center & Department of Psychiatry, University of British Columbia, Vancouver, Canada; University College London, United Kingdom

## Abstract

A common theoretical view is that attractor-like properties of neuronal dynamics underlie cognitive processing. However, although often proposed theoretically, direct experimental support for the convergence of neural activity to stable population patterns as a signature of attracting states has been sparse so far, especially in higher cortical areas. Combining state space reconstruction theorems and statistical learning techniques, we were able to resolve details of anterior cingulate cortex (ACC) multiple single-unit activity (MSUA) ensemble dynamics during a higher cognitive task which were not accessible previously. The approach worked by constructing high-dimensional state spaces from delays of the original single-unit firing rate variables and the interactions among them, which were then statistically analyzed using kernel methods. We observed cognitive-epoch-specific neural ensemble states in ACC which were stable across many trials (in the sense of being predictive) and depended on behavioral performance. More interestingly, *attracting* properties of these cognitively defined ensemble states became apparent in high-dimensional expansions of the MSUA spaces due to a proper unfolding of the neural activity flow, with properties common across different animals. These results therefore suggest that ACC networks may process different subcomponents of higher cognitive tasks by transiting among different attracting states.

## Author Summary

For understanding how neural processes give rise to cognitive operations, it is essential to understand how aspects of the underlying neural network dynamics reconstructed from neurophysiological measurements relate to behavior. For instance, different actions may be represented by neural states characterized by stable population patterns to which activity converges in time, called *attractors* in the language of dynamical systems. However, experimental demonstrations of neural attractors associated with cognitive entities have been rare so far. One problem may have been that in behaving animals, in-vivo one can access only a relatively small fraction of the total number of neural units comprising the whole system, even with modern multiple single-unit (MSU) recording techniques. Therefore, the neural activity dynamics are necessarily projected from a very high-dimensional into the empirically accessible much lower-dimensional space in which attractor properties may be lost due to ambiguities and entanglement in the flow of trajectories. In the present study, principles from nonlinear time series analysis and statistical learning are applied to MSU recordings from the rat's prefrontal cortex during decision-making tasks. By expanding the empirically accessed neural state space (semi-) attracting properties of neural states corresponding to cognitively defined task-epochs became apparent, in line with many neuro-computational theories.

## Introduction

To fully understand how neural processes give rise to cognitive operations, it is essential to reconstruct the underlying neural network dynamics from electrophysiological or neuroimaging measurements in relation to behavior. A common theoretical idea is that these *dynamical properties* of the nervous system, like the convergence of activity to specific stable population patterns (attractors), are what ultimately implement the computational operations that link inputs to outputs [1]–[6]. For instance, different attracting states may represent different active memories or cognitive entities, and movement between these states may correspond to the recall of a memory sequence or the execution of a behavioral or motor plan. Attractor states as a basis for cognition received particular attention in the context of working memory [2], [4], [7]–[9] and decision making tasks [5], [10]–[12].

Especially in recent years, along with the advances in multiple single-unit recording techniques [13], there has been a dramatic rise in the attempts to reconstruct cognitively relevant aspects of the population dynamics. Many of these relied on methods from multivariate statistics and machine learning (as reviewed in [14], [15]). These studies gave a number of valuable insights into mechanisms of neural information processing like the information content of the transient dynamics connecting steady states [16], [17], the representation or processing of stimuli by reproducible sequences of states [18], or the sudden nature of transitions among representational states during learning [19]. Several experimental studies also suggested that spatial representations in the rodent hippocampus [6], [20]–[22] or olfactory representations in zebrafish [16], [23] may have attractor-like properties with sometimes stochastic transitions among them [24], [25]. In these studies, attractor states were indicated by discrete switches in the population activity patterns eventually attained (after some transient) when stimulus parameters were continuously varied. Strictly speaking, however, these studies did not attempt to explicitly demonstrate a convergent flow of neural trajectories (as sometimes pointed out by the authors themselves, [23]), as another important signature of attracting states. Moreover, they mostly focused on (stimulus-driven) sensory or spatial representations rather than on presumably intrinsically-driven higher cognitive processes. In addition, since most of these previous approaches worked directly in the space of observed variables, i.e. the recorded units' firing rates or spike times, they could potentially miss some important structural details of neural space organization, especially in high-noise situations, as they try to infer the dynamics of a large complex system by selecting only a few of its dimensions (recorded neurons). Thus, experimental evidence for the hypothesis that higher cognitive processes proceed by moving among attracting states is still sparse.

Here we combined and adapted two approaches well established in statistical learning theory [26], [27] and nonlinear time series analysis [28], [29] in an attempt to move beyond some of the limitations that could arise in previous analyses of electrophysiological data. These methods were applied to multiple single-unit recordings from the rat anterior cingulate cortex (ACC) during a complex memory-guided decision making task in a radial arm maze (Figure S1). The ACC is assumed to play a key role in higher-level cognitive processes like monitoring of behavior [30], processing error feedback [31], making choices [32] and dissecting task structure [33]. Thus, the ACC is a brain area with complex intrinsic dynamics and computational properties that presumably demand a sophisticated multivariate analysis to much larger degree than comparatively simpler early sensory systems (e.g. [16], [23]). The present analysis was designed to be more sensitive to potential state space structure, suggesting previously unrecognized convergence properties of ACC neural ensemble states associated with cognitive processing steps and stable across multiple trials.

## Results

### Neural state space reconstruction: Motivating the approach

**Figure 1:**
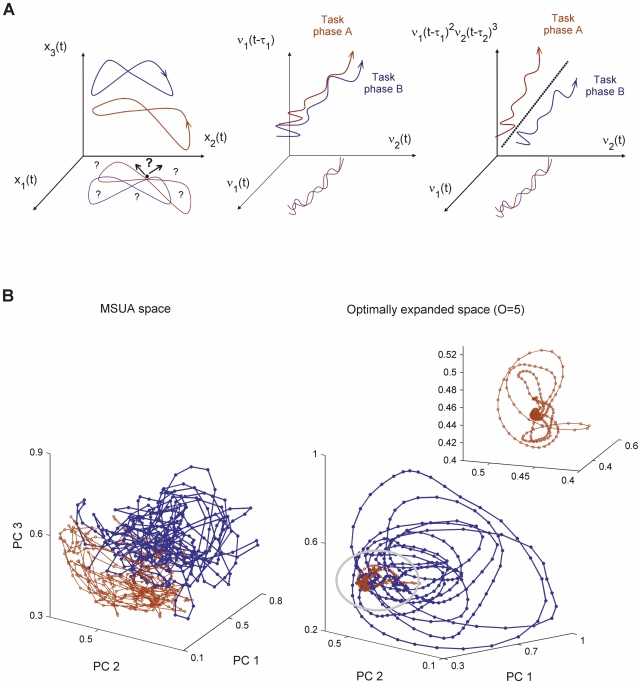
Unfolding trajectories by expanding state space dimensionality. **A.** Left: In this schema, the two-dimensional reconstruction of a three-dimensional dynamical system in the plane (*x*_1_, *x*_2_) causes two trajectories to intersect with themselves and with each other multiple times (as indicated by the dots). At each of these intersection points, the flow of the system (the change of activity in time) is not uniquely defined as indicated by the arrows and question marks. However, such a unique determination of flow would be important for assessing, e.g., the convergence of trajectories. Center: A potential solution: While it may not be possible to discriminate between two trajectories within a two-dimensional plane spanned by the firing rates of two neurons, (ν_1_(*t*), ν_2_(*t*)), adding a third axis containing an appropriate time delay for one of the units permits to fully disentangle the two trajectories. Right: High-order products of delayed firing rates, e.g. ν^2^_1_(*t*-τ*_1_*) ν^3^_2_(*t*-τ*_2_*), further amplify the trajectory separation already achieved through the delays. Thus, dimensions missing from the original space can be substituted by new axes formed from the measured variables. **B.** Three-dimensional projections obtained by Principal Components Analysis (PCA) for a single trial (#1) of rat #1 (see text). Brown curves represent the training and test phases, and the dark blue curve indicates the delay period in the radial arm-maze task shown in Figure 2A. Left: PCA reduction of the MSUA space. Right: Kernel-PCA reduction of the expanded space containing higher-order activity products. The neural trajectories intermingled on the left become nicely unfolded on the right.

A state space is a coordinate map spanned by all relevant dynamical variables of a system (e.g. the membrane voltages or firing rates of neurons). A single (vector) point in this space represents the whole state of the recorded neural system at a given point in time (e.g. the current firing rates of all neurons), while a trajectory in this space charts how its state changes over time. Most computational theories of the brain work by linking geometrical objects in these spaces (e.g. attractors) and the temporal evolution of neural activity (the trajectories) to specific computational and cognitive functions (e.g. [2], [4], [34]–[37]). However, inferring the dynamics of a large complex system from experimental data by selecting only the observable dimensions (recorded neurons) can lead to incorrect conclusions [28], [29]: Neural trajectories may not be sufficiently “unfolded”, i.e. may follow apparently convoluted patterns where they frequently “intersect” themselves and exhibit ambiguities with regards to their direction of flow (Figure 1A, left). This is due to the fact that other dimensions along which the flow would have been disambiguated are missing (e.g. the third axis in Figure 1A, top left; [28], [38]). Thus, a state space construed solely from the activities of the simultaneously recorded units (termed multiple single-unit activity, MSUA, space in the following) is not guaranteed to properly represent the geometry of the underlying dynamical system's attractors.

A potential solution to this problem was provided by time series embedding theorems [28], [38] which demonstrated that the structure of the underlying attractor dynamics could be fully recovered (under ideal, noise-free conditions) if the dimensionality of the space is expanded by adding a sufficient number *p* of time-lagged versions ν(*t-τ_i_*) of the present observations ν(*t*) as new variables to the space, where the time lags *τ_i_* are determined such that these new variables do not contain redundant information with respect to the original MSUA axes, i.e., are only weakly correlated with them (Figure 1A, center). In principle, the optimum number of delay axes is constrained by the dimensionality of the underlying attractor of the system [28]. Unfortunately, however, due to the sparseness of the MSUA spaces and the noise levels in these data it cannot be reliably computed. Moreover, given that for neural systems the true dimensionality could be (much) higher than the number of dimensions one has experimentally access to, the number of time lags required for a statistically optimal disambiguation of trajectory flows may be so high that it cannot be accommodated by the (experimentally) limited length of the time series (Materials and Methods).

Therefore, it may be necessary to consider also other types of state space expansion that allow to effectively discern the neural dynamics associated with different cognitive events. Adding interactions between units' firing rates as dimensions to the space seems a particularly suitable choice since neuronal cross-correlations have often been postulated to play an important role in cognitive processes (e.g. [39]–[43]). From a mathematical point of view products of neural firing rates would correspond to terms of a multinomial basis expansion frequently employed in statistical classification procedures [27]. Hence, such an expansion would have both a neuroscientific meaning and a theoretical foundation. Therefore, in our approach the delay-coordinate (DC) map of the MSUA space (DC-MSUA space) is further expanded by adding pairwise and higher order cross-products of the recorded units' firing rates, up to some order *O*, as new dimensions. For example, an expanded state space of *3^rd^* order will contain all the original MSUA axes, plus time-delayed versions of the firing rates of all *n* recorded units, ν_1_(*t*-τ*_1_*), ν_2_(*t*-τ*_2_*),…, ν*_n_*(*t*-τ*_n_*) as well as new axes corresponding to third order products like ν_1_(*t*-τ*_1_*) ν_2_(*t*-τ*_2_*) ν_3_(*t*-τ*_3_*) or ν_1_(*t*-τ*_1_*)^2^ ν*_3_*(*t*-τ*_3_*). Vectors in these high-dimensional spaces will be denoted by **Φ**(t) – each such vector corresponds to a specific (spatio-temporal) pattern of neural firing rates and firing rate correlations up to the order set by the expansion. Since the dimensionality of such spaces can be extremely high, specialized algorithms (so-called *kernel*-methods [44]–[46]) were used for the statistical analyses, as discussed below. As illustrated in Figure 1A (right), adding these cross-product terms can help to further disentangle neural trajectories by amplifying small differences present in the DC-MSUA space.

Why this 2-stage process in expanding the original MSUA space? If trajectories in the originally recorded MSUA space are already nicely disentangled and noise levels are very low, no further expansion may be necessary. However, many of the simultaneously recorded neurons may fire very sparsely, or may otherwise be non-informative about the system's dynamics, or there may simply not be enough of them which access “sufficiently different aspects” of the system's dynamics. Adding delay coordinates (with delays chosen such as to minimize cross-correlations among the firing-rates of different neurons, see Materials and Methods) will increase the amount of information about the neural dynamics captured by the space by removing ambiguities in the neural flow which may occur in the MSUA space (Figure 1A, center). Adding product terms, on the other hand, may not add further information about the dynamics to the space (although it may make information contained in neuronal correlations explicitly accessible), but it will help to pull trajectories apart and thus enhance task-related differences in the activity flow in situations of high noise (Figure 1A, right; see also Materials and Methods). It may also take care of the fact that putative attractor geometries may be highly nonlinear structures that are not easily captured by linearly separating hyperplanes. Hence, by combining these two types of expansion we arrive at a space which should be both, more informative due to the addition of delay coordinates, and at the same time “less noisy” and more apt for detecting nonlinear structures. Here we show that the identification of ensemble dynamics for different animals and behavioral performance levels will, in general, indeed significantly improve by combining both types of expansion.

As an example, Figure 1B shows a single *trial* of an animal performing a higher cognitive task explained in the next section. A type of principal component analysis (PCA) suitable for very high-dimensional *O^th^* order spaces, termed *kernel*-PCA [47] (for *O* = 1 equivalent to conventional PCA), was used to visualize the neural dynamics in the 3 most variance-explaining dimensions. While for both the MSUA and *O* = 5 spaces the two illustrated task phases (blue and red dots in Figure 1B) can be clearly discerned, the actual trajectories (the lines connecting the dots) are quite entangled in the MSUA space but are nicely unfolded for high-order expansion spaces, exposing attracting orbits and properties of the two task phases (Figure 1B; see also Video S1).

### Visual analysis of task-epoch specific population states

**Figure 2:**
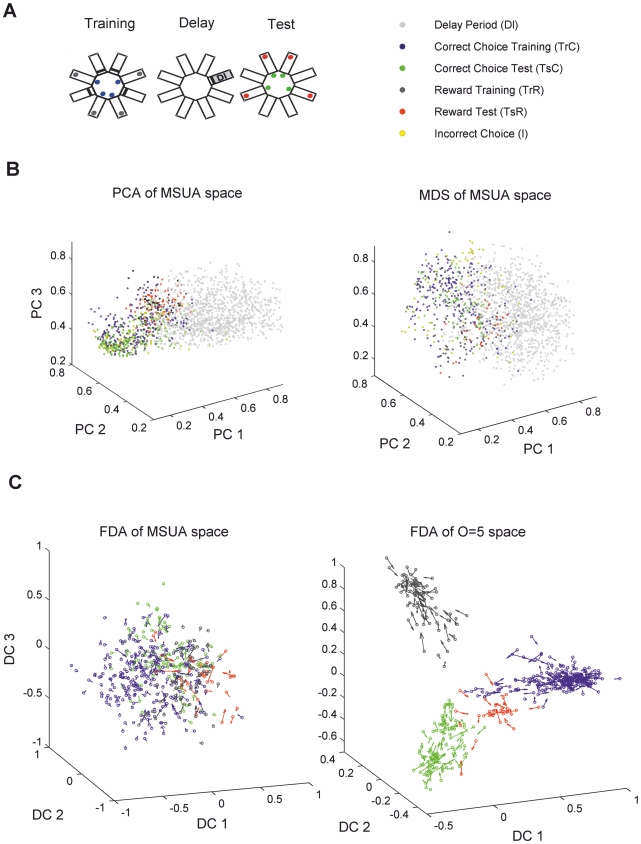
Visualization of task-epoch-specific dynamics. **A.** Schema of the delayed win-shift radial arm maze with the definition of separate task epochs (see Materials and Methods for exact definition). **B.** Three-dimensional projections of the MSUA space combining trials 1 to 5 of animal #1, obtained by PCA (left) and by Multi-Dimensional Scaling (right). **C.** Three-dimensional projections obtained by a Fisher Discriminant Analysis (FDA) of the training and test choice and reward epochs (multiple classes, centered and normalized for clarity) with the flow field (velocity vectors) indicated by arrows, i.e. these vectors give the magnitude and direction of change of the projected neural activity. Left: MSUA space. Right: Expanded *5* ^th^ order space (using kernel-FDA). As in **B**, trials 1–5 of animal #1 were combined for this graph.

The techniques introduced above were used to analyze MSU recordings obtained from the rat ACC (Figure S1) while the animals were situated in a radial-arm-maze decision-making task with temporal delay (Figure 2A). This task is considered to be ecologically valid in the sense that it mimics key aspects of rats' natural foraging, food-hording, and retrieval behavior (e.g. [48], [49], [33]). The entire time on task was divided into six epochs with differing cognitive demands as illustrated in Figure 2A (see Materials and Methods for precise definition of the cognitive epochs). Two data sets were available for the present analyses: 1) Three animals recorded for up to 15 trials solely for the purposes of the present study. From these, only trials with good performance were selected ( = less than 3 test phase errors; median errors across all trials were 1, 0.5 and 2 for respectively for each animal), with an error defined as re-entrance into an arm from which food was already retrieved. 2) Six animals recorded for one or two trials from a previous study [33], which will be used to further confirm the results obtained with the “multiple-trial animals” and to conduct an explicit comparison of high (<2 errors) vs. low (>4 errors) performance trials. Average trial duration (±SEM) was 159.3±19.7 s across all trials and animals. With a standard binning for the spike density estimates of 0.2 s, this resulted in an average of 797±99 firing-rate vectors per trial (see further below for a discussion on data size effects).

To provide a direct comparison with previous approaches for constructing neural state spaces, Figure 2 shows three dimensional projections obtained in different ways from the first five trials of one of the multiple-trial animals which performs the task with less than three errors per trial. Consistent with our previous observations [33], the MSUA space shows a visually apparent segregation among the different task epochs (indicated by the color-coding), using either PCA (Figure 2B, left) or multi-dimensional scaling (MDS; Figure 2B, right) for the 3-dimensional reconstruction.

Figure 2C shows the same data projected into a 3-dimensional space using a Fisher discriminant analysis technique (FDA; see e.g. an application to MSUA spaces in [19]). Like PCA, FDA amounts to just a linear transformation of the original variables. However, unlike PCA, the directions sought are such that the differences between group means are maximized while at the same time within-group jitter is minimized along them (for the *O^th^* order higher-dimensional spaces we used a regularized kernel-FDA which is equivalent to a standard (regularized) FDA for *O* = 1 [50]; see Materials and Methods and Text S1). The figure displays the *flow field* in addition to the data points, i.e. the speed and direction of movement of the neural population state at each time bin (computed as the difference between temporally consecutive vector pairs). While the flow field in the FDA-reduced original MSUA space may appear relatively disordered (Figure 2C, left), in the expanded space (Figure 2C, right) a consistent movement into each of the task related clusters at points far from any cluster center appears to occur (as will be statistically confirmed below). In summary, these 3-dimensional visualizations seem to suggest that different cognitively defined task epochs are associated with different population states which exhibit attractor-like properties (convergence of flow), a phenomenon that becomes apparent only after expanding the spaces to sufficiently high dimensionality using the techniques outlined in the previous section.

We stress that, in principle, expansion of spaces to much higher dimensionality is a well-known technique in statistical classification approaches to improve the linear separability of classes [27]. However, a serious statistical issue with such approaches is the potential problem of “over-fitting” the data: For instance, *n*+1 points can always be perfectly linearly separated in a *n*-dimensional space, even if their configuration is purely random. To circumvent this problem, two approaches which are standard in statistics (e.g. [46]) and machine learning (e.g. [44]) were employed here: First, a regularization term (fixed throughout the study; Eq. S3 in Text S1), which penalizes model complexity and thus reduces the *efficient* dimensionality of the fitted classifier (typically way beyond the nominal dimensionality), was included in the optimization criterion for the kernel-FDA. The technique of cross-validation (e.g. [44], [46]) is used in the next section for deriving this regularization term and the expansion order optimal for across-trial predictions (Materials and Methods). Over-fitting would imply poor generalization to new data sets not used for fitting the classifier, i.e. a high out-of-sample prediction error across trials. Second, the performance of the classification statistics on the original data was compared to bootstrap data in which the relation between neural population vectors and cognitive-class labels has been randomized. Such bootstrap samples have to be devised carefully such that they retain features of the original time series (like their temporal autocorrelations) which are not necessarily related to task-imposed structure, as explained in the sections to follow.

### Stability of task-epoch specific states across trials

For determining the optimal state space we assessed whether the assignment of population-interaction patterns to task epochs could be correctly predicted in a test set of trials based on information obtained solely from a non-overlapping training set of trials, or, from another perspective, how stable the task-epoch-specific clusters in *O^th^*-order expansion space are across multiple trials. To these ends, state spaces were reconstructed exclusively from the first set of 4 to 8 well-performed trials, and data points from the (non-overlapping) set of the last 4–8 well-performed trials were projected into this space (“forward predictions”). Vice versa, “backward predictions” from the last to the first trials were also obtained. If the neural dynamics remain largely invariant across multiple trials, then vector points on any subsequent trial should fall into the same clusters derived only from the first few trials. This analysis was performed for any pair of task epochs using the most discriminating direction as obtained by kernel-FDA within the expanded high-dimensional spaces. Assuming that the projections of the *O^th^*-order population vectors from any two task epochs onto this maximally separating direction are normally distributed (which will almost inevitably be the case due to the central limit theorem, as the projections are sums of many random variables), for each population pattern **ν**(*t*) the probabilities *P*(**ν**(*t*)|*C*_1_) and *P*(**ν**(*t*)|*C*_2_) that it comes from one task-epoch or the other can be evaluated. Assigning population vectors to task epochs based on these probabilities yields a segregation error (SE) for each pair of task epochs defined as the relative number of misclassified population patterns **ν**(*t*) (see Materials and Methods for discussion of further advantages this brings over other kernel-based approaches). By chance this misclassification rate will be 50% since we fixed the *prior* probabilities *P*(*C*_1_) and *P*(*C*_2_) at 0.5 for any pair of epochs, such that the results would not be biased towards the longer-lasting epochs. Note that all time bins (population vectors) from a given task epoch class were entered into this analysis, regardless of whether they came from the same or from different trials.

**Figure 3:**
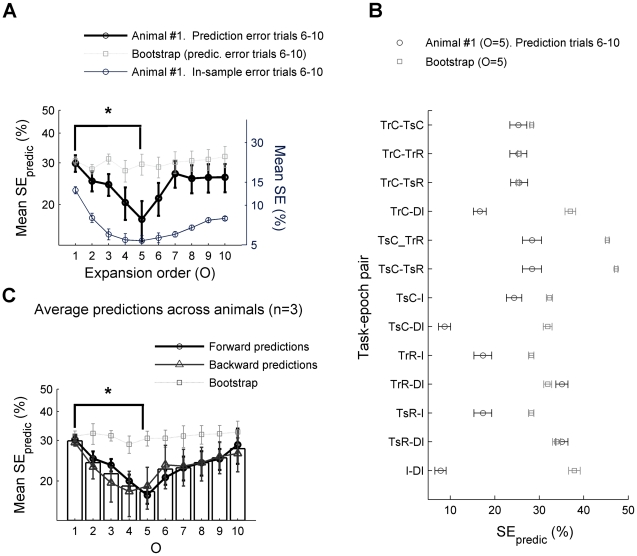
Out-of-sample (across trials) predictability of the task-epoch-specific organization of population activity. **A.** Statistical analysis of across-trial predictability for animal #1. The predicted SE (SE_predic_) is obtained by first constructing a classifier for each pair of task epochs based on a regularized version of Fisher's discriminant criterion exclusively from the first few trials, and then applying it for assigning activity vectors from the last few trials (the test set) to task epochs. SE_predic_ values averaged across all task-epoch pairs (error bars = SEM) reach a minimum at the rate-interactions order *O* = 5 and are significantly lower than those obtained for matched bootstrap data (one-sided non-parametric test at p = 0.01). In contrast, the DC-MSUA space (*O* = 1) does not reveal this predictive structure (p>0.1). Note that chance level is 50% here since a-priori probabilities were set to 0.5 for each pair of task epochs. Reward epochs were excluded from the comparisons due to too few data points. *y-axis* scale is logarithmic in plots A and C. The asterisk indicates a significant difference in the comparison *O* = 1 vs. *O* = 5 for the original data (t-test, Wilcoxon ranksum test, n = 6, both p<0.05; normality assumptions valid according to Lilliefors and Chi-square tests, p>0.12). The regularization penalty was selected such that it provides the minimum SE_predic_ for different orders *O* for this particular animal, and then was fixed for all other analyses (see Figure S3 for results obtained with different values of the regularization parameter used in the kernel-FDA). **B.** Individual comparisons for all task-epoch pairs for the *O* = 5 space. **C.** Mean SE_predic_ averaged across all three recorded animals, attaining a significant minimum at the rate-interactions order *O* = 5 (Wilcoxon rank-sum test, n = 3 animals, p<0.05). Both forward (from the first to the last set of trials) and backward (from the last to the first set) are shown. Detailed results for animals #2, 3 are shown in Figure 4.

For checking predictability across trials, the crucial aspect now is that the optimal discriminant direction was solely obtained from the first (or last) couple of (reference) trials, and then fixed and used for out-of-sample predicting the corresponding misclassification rate SE_predic_ (for “predicted SE”) of population interaction patterns to task-epochs for the non-overlapping set of last (or first, respectively) prediction trials (see Materials and Methods for more details). To evaluate the significance of the observed SE_predic_, bootstrap data were constructed by randomly shuffling stretches of the **ν**(*t*) vector time series that retained entire trajectories form a given specific task epoch, i.e. each bootstrap replication preserved all temporal autocorrelations up to the length of the relevant task epochs. Consistent with the visual displays presented above, for *O*∼5 SE_predic_ was significantly lower (p<0.01) in the original as compared to the bootstrap data (Figure 3A; see Figure S2 for a schema on bootstrap construction). Note, however, that SE_predic_ for the bootstraps is also less than what would be expected by chance, i.e. <0.5, such that prediction *accuracy* in the bootstraps is above chance level. This is because the bootstraps retain original auto-correlations as indicated above, which by themselves may induce some state space clustering, irrespective of task-epoch membership. Surprisingly, in contrast to the case *O*∼5, for *O* = 1 (i.e., within the DC-MSUA space) predictability across trials was not significantly better in the original than in the bootstrap data. Thus there does not seem to be sufficient information in the lower-dimensional state spaces to allow prediction of population pattern assignments across trials. Rather, given the experimental noise and the potentially nonlinear state space structures, neural interactions have to be included to establish stable associations between task epochs and population patterns, or, in other words, further trajectory separation beyond the one achieved by delay-coordinates is indeed necessary to reveal across-trial stability. Specific comparisons for each pair of task epochs are shown in Figure 3B. Finally, for *O*>5 predictability starts to deteriorate again. Hence, it seems that there is a maximum order of activity products which would be required to optimally resolve task-epoch-related structure in the neural state spaces, a finding consistent across the different data sets studied (Figure 3C). We emphasize that this result does not imply that neural activity interactions up to some precise order (*3^rd^–5^th^*) are important– it only shows that below or above a certain expansion order *generalization* performance degrades, which can be the case for purely statistical reasons (i.e., simply because there are too few data or too few simultaneously recorded neurons to reliably estimate the optimum order of interactions).

**Figure 4:**
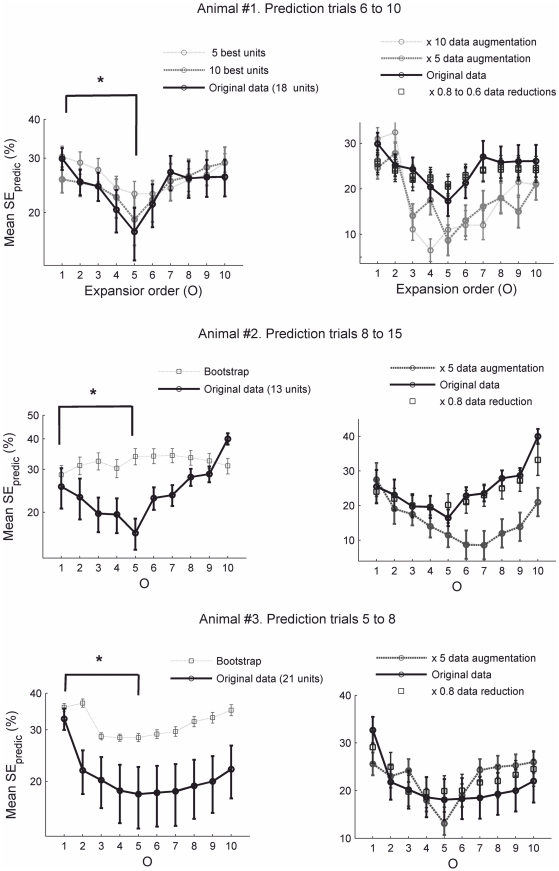
Robustness of across-trials predictability of task-epoch-specific organization of population activity. Plots show results for different ACC networks (different animals), numbers of recorded units, and sample sizes (numbers of trials). **A.** Left: Statistical analysis of SE_predic_ for different numbers of selected units from animal #1. Right: Analysis of SE_predic_ for animal #1 for different numbers of data points obtained by artificially augmenting or decimating the original data set (by either bootstrapping the original data or randomly removing vectors from it). **B.** Same for animal #2. **C.** Same for animal #3. Note that optimal prediction always occurs around similar high-orders of rate interactions as for animal #1 (Figure 3). Asterisks indicate significant differences for the comparisons indicated (Wilcoxon rank-sum test, n = 6, p<0.05 for both animals #2 and #3; nonparametric tests were used because Lilliefors [p<0.003, 0.04] and Chi-square [p<0.003, 0.01] tests indicated that the data significantly deviated from normality, thus violating the assumptions for parametric testing).

On the other hand, the optimal orders we obtained do not seem to be completely arbitrary (in the sense of being determined purely by the number of data points and recorded units): First, similar optimal orders were also observed for the other two animals (Figure 4) which differed in the number of recorded units (18, 13 and 21, respectively) and the size of the training and prediction sample sets (5, 8 and 4 trials, respectively). Second, we performed additional controls by including subsets of neurons of differing size (Figure 4, upper left) and by artificially augmenting or decimating the data sets in a way that preserved the original distributions (Figure 4, right). Hence, we conclude that there is an organization of task-related population interaction patterns predictable across many trials which is optimally revealed by expanding the MSUA space by taking higher orders of activity interactions into account.

### Relation of state space structure to behavioral performance

**Figure 5:**
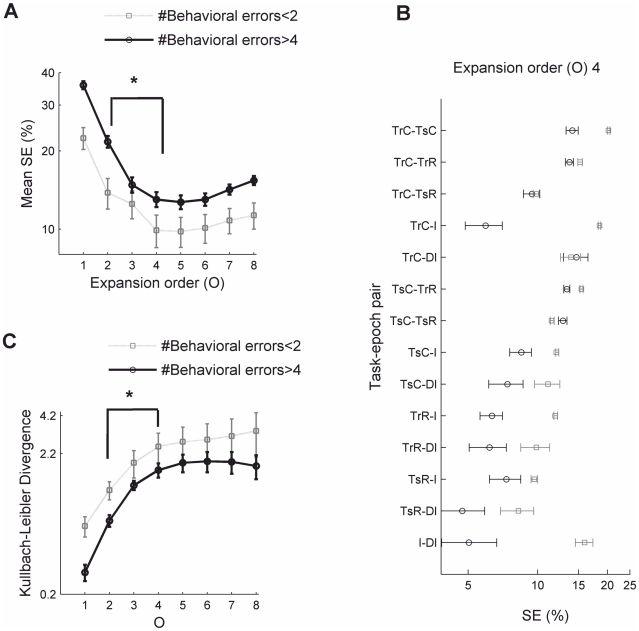
Statistical analysis of task-epoch separation in state spaces for the many-animal single-trial data set. Black solid curves from 8 trials in which animals performed with less than two incorrect arm choices, gray dotted curves from 8 trials where more than four incorrect arm choices were made. **A.** Task-epoch mean segregation errors (SE) for the good (grey) and bad (black) performance groups averaged across all task-epoch pairs (n = 14, error bars = SEM). Asterisks indicate significant differences for the comparisons indicated. For high-performance animals, a two-tailed non-parametric Wilcoxon rank-sum test was used (n = 14, p<0.04), as data significantly deviated from normality (two-sided Lilliefors test, p<0.03; Chi-square test, p<0.01). For the low-performance group, normality held (Lilliefors test, p>0.44, 0.41), and the comparison between *O* = 2 and *O* = 4 conditions is highly significant using either a t-test (p<0.0001) or Wilcoxon ranksum test (p<0.0001). Comparisons between low- and high- performance groups are also significant for *O*>3 (n = 14; p<0.03, Wilcoxon ranksum test). **B.** Individual task-epoch-pair comparisons. **C.** Kullback-Leibler distance between task-epoch distributions averaged across all task-epoch pairs for high- (black) and low- (gray) behavioral performance trials (asterisk and error bars as in **A**). Task-epoch distributions chart the probabilities of the animal being in a task-epoch *C* given a population activity vector **ν**(*t*), i.e. *P*(*C*|**Φ**(*t*)). See Materials and Methods for more details.

In a previous study [33] we had compared animals performing well on the task to animals which committed a lot of behavioral errors. We observed that in animals performing poorly state space segregation (task-epoch-dependent clustering) was generally comprised compared to trials on which only few (0 or 1) errors were committed. Here we re-addressed this issue using the methods developed above (Figure 5). Data from 8 trials (coming from 4 different animals) performing with less than two errors ( = “good performers”) and 8 trials (coming from 5 animals) with more than four errors ( = “bad performers”) were used. These two groups of trials were combined into two separate data sets for analysis (termed “single-trial” datasets). This works since the basic structure of the cognitively-defined classes was the same for all animals, i.e., the task obviously was the same for all animals, and population patterns specific for different task episodes like choices, rewards, or the delay phase, were a common feature of ACC activity. Since only a single trial with electrophysiological recordings, however, was generally available from each of these animals, results were cross-validated by removing each single one of the animals from the data set in turn (i.e., a jackknife validation [51]).

Consistent with our previous observations [33], discriminability in the MSUA space is significantly worse (Wilcoxon rank-sum test T_13_ = 113, p<0.05) for “bad performers” (Figure 5A, dark curve for *O* = 1) when compared to “good performers” (Figure 5A, gray curve for *O* = 1). However, as Figure 5A shows, for *both* groups discriminability significantly increases just up to expansion orders of about 5, i.e. the segregation error (SE) as defined further above (computed from FDA with the same regularization as above, see Materials and Methods) significantly decreases (Wilcoxon ranksum tests, p<0.03; see details in Figure 5 legend). Thus, as the maximum order *O* of the reconstructed state space is increased, cognitively relevant features of the neural dynamics are increasingly better resolved to the extent that an organized dynamics becomes evident even in situations where previous methods had failed (see [33]). However, as for the multiple-trials data analyzed in the previous section, SE for *O*>5 grows again for both groups (Figure 5A), suggesting once again that there may be a maximum order of activity interactions for which trajectories are optimally resolved.

Finally, and again consistent with previous results [33], although SE decreases for both groups, there still remains a significant difference between the low and the high performance groups even for *O*>3 (Wilcoxon test, p<0.04), confirming that still some of the state space organization is corrupted in bad performers. Detailed task-epoch comparisons are shown in Figure 5B. Similar results were obtained with information-theoretic measures of task-epoch segregation like the relative entropy (Kullback-Leibler divergence, e.g. [44]) between the conditional probability distributions of task-epochs given a specific firing-rate vector (Figure 5C; see Materials and Methods section). Moreover, further control analyses indicated that results are not significantly altered by using state spaces constructed by using different types of expansion, other classification criterions, or other smoothing parameters for the spike trains (as shown in Figure S3).

### Convergence towards task-epoch-specific neural ensemble states

**Figure 6:**
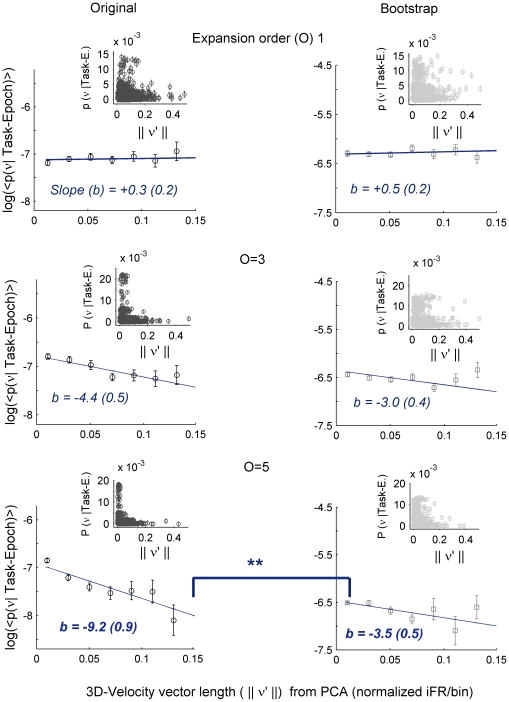
Convergence of trajectories as assessed from 3-dimensional projections. As an approximate measure of convergence to task-epoch states the likelihoods of correct classification of population vectors **ν**(*t*) into task-epoch sets, i.e. *p*(**ν**(*t*)|*Task-Epoch*) were charted as a function of the amplitude of the velocity vector in the 3-dimensional PCA projection (determination of velocities directly in the *O^th^*-*order* spaces is very unreliable due to their high dimensionality; e.g. [44]). In other words, these graphs give the probability density of correct assignment of a neural activity pattern to the right task epoch, or correct-class-likelihood, as a function of the rate of activity change at this point (normalized values across all vectors). Class-likelihoods were based on Bayes-optimal classifiers within the high-dimensional *O^th^*-order spaces and were assessed on test sets of trials (as explained in Figure 3), i.e. refer to out-of sample predictions. Graphs are for increasing rate-interaction orders *O* from top to bottom. Left: original data; right: bootstrap data (inversion of time). Error bars of insets give 99% confidence intervals. As *O* increases, lower velocities are associated with higher likelihoods of correct classification, indicating that the neural system dynamic slows down as it approaches the center of such putative attracting sets (see discussion in the main text). Linear fits to the averages of *log*(*P*(**ν**(*t*)|*Task-Epoch*)) versus velocity across the 20 bins into which the x-axis was divided (RMS error of fits <1% of the geometric mean; numbers *b* refer to the slopes of the fits) revealed that differences in slope between the original and bootstrap data were highly significant for *O* = 5 (p<0.006, t-test, n = 20) but not *O* = 1 or *O* = 3. Data shown are for multiple-trial datasets. Insets give the full distributions of data points.

The most interesting aspect of the present methodological approach is that it permits to examine the *flow* of neural trajectories during performance of a cognitive task, dynamical properties that may not be well accessible in the unprocessed representation of MSU activity as demonstrated in the previous sections (Figure 3B, left). Here we analyzed the attracting behavior suggested by the three-dimensional visualizations more systematically. First, a simple statistical approach was taken. Activity flows were evaluated in the low-dimensional kernel-PCA projections of task epochs, since velocity vectors cannot be reliably obtained in the extremely high-dimensional expanded spaces (for similar reasons for which we used kernel methods before; see Figure S4 and Text S2 for further discussion). Figure 6 displays the speed of movement at each data point in these projections as a function of the likelihood of a population pattern given the task epoch to which it belongs, i.e. *p*(**ν**(*t*)|*correct task-epoch classification*), evaluated using FDA in the high-dimensional *O^th^*-order spaces for the prediction set of trials (see Figure 3). If the task-epoch states have indeed attracting properties, one would expect that vector points which exhibit little movement should have a high likelihood of correct classification, reflecting the fact that these points should be found close to the cluster centers. Consistent with the idea that in low-order spaces trajectory flows should appear convoluted and disordered, for *O* = 1 velocities were evenly distributed across all regions of the state space, i.e. the velocity of movement of the neural state was largely independent of the likelihood of correct classification (Figure 6, left-top; *O* = 1). In contrast, for higher-order expansions the likelihood of correct classification rapidly falls off as the speed of neural state changes increases (Figure 6, left-bottom; *O* = 5), confirming that regions where trajectories move quickly are on average far from the cluster centers.

Although these results are suggestive, they by themselves do not conclusively rule out alternative explanations unrelated to the potentially attracting nature of the task-specific ensemble states, e.g. the tendency of extreme values to be followed by values closer to the mean simply by laws of probability (“regression to the mean”), auto-correlative properties of the time series, or by systematic deformations of the flow field induced by PCA. To statistically control for such alternatives, we performed a bootstrap test. The right column of Figure 6 shows results from the same analysis as performed on the bootstrap data when the temporal sequence of binned firing rates was inverted for all neurons within task-epochs. Therefore, task-epoch-specific lengths are preserved, but any causal relationships in the original time series are destroyed. For *O* = 1, the correct classification likelihood as a function of velocity behaves similar for bootstrap and original time series, but at higher expansion orders the fall-off of correct classification likelihood with vector velocity is significantly less steep in the bootstrap than in the original time series (paired *t-test* between the two slopes, p<0.001 for *O* = 5, see Figure 6 caption) as demonstrated by the linear fits to the log-linear graphs. In summary, different cognitively defined task epochs may potentially act as attracting states of the neural dynamics, i.e. regions of state space towards which all trajectories tend to converge with high likelihood and within which they remain bounded for some time.

**Figure 7:**
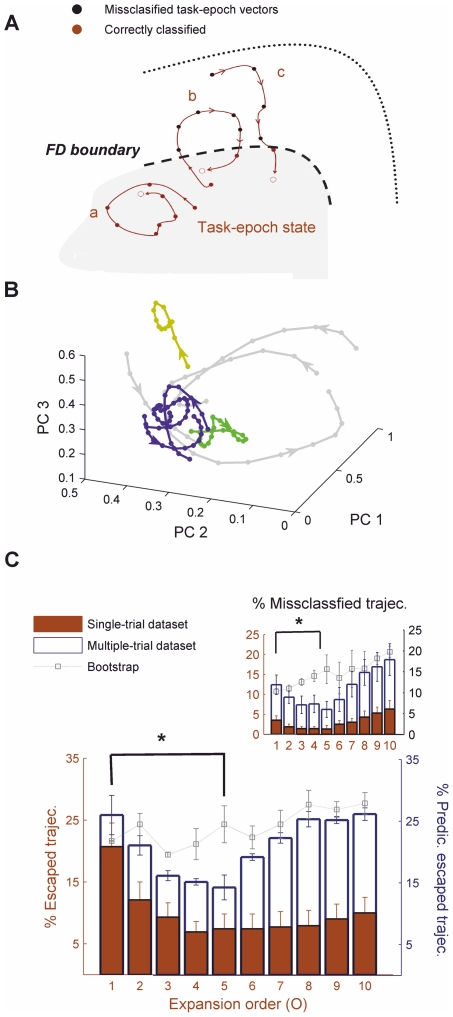
Quantitative assessment of the attracting behavior of task-epoch specific ensemble states within the full high-dimensional spaces. **A.** Schema illustrating different types of trajectories which would constitute evidence for an attracting region defined by the task epochs: Trajectory “a” is completely confined within the task-epoch state, trajectory “b” leaves the task-epoch state (for instance, due to perturbation by noise) and then quickly converges back to it, and trajectory “c” is rapidly attracted into the task-epoch state. Black dots in the figure highlight incorrectly classified firing-rate vectors. If only trajectories of types a-c were present, this would strongly suggest that the task-epoch states are indeed attractors. This condition is formally evaluated in C. **B.** Examples of convergent trajectories, cycling within or returning to the task-epoch states, in the 5^th^-order expanded spaces (corresponding to different trials of the task). **C.** Percentage of trajectories which escape from task-epoch states for the single-trial data sets (red) and as predicted across trials for the multiple-trial data sets (white) within the full high-dimensional *O^th^*-order spaces. A total of ∼20 trajectories was available for each task-epoch specific state during the prediction set of trials. The absence of any trajectories escaping from a bounded region of state space (as defined by the task epochs), i.e. if only trajectories of types a-c were present, would suggest the existence of a basin of attraction which is stable across trials. Asterisk indicates significance for the comparison *O* = 1 vs. *O* = 5 (nonparametric Mann-Whitney-U test, p<0.05). Inset: A weaker condition (attracting set condition) will be met if trajectories escaping from the task-epoch set still remain within a trapping region [70]. The graph gives the average percentage of trajectories violating this attracting set definition. Note that task-epoch specific states were defined by narrow (∼1 s) temporal windows around the relevant event such that the majority of available data points are not included in the definition of any one of these states. Hence, the attracting properties revealed here are not just a trivial consequence of a very broad definition of the neural states.

While this analysis suggests attracting behavior related to the task epochs, it was performed on a three-dimensional representation in which velocity vectors could still be reliably determined. We therefore next sought to precisely quantify within the *full high-dimensional spaces* to which degree the (mathematical) conditions defining attracting states were met in the empirical data, with the statistical analysis based on the task-epoch boundaries defined previously. As the definition of these boundaries did not include any knowledge about putative attractor states, there is no a-priori reason why there should be strong convergence over time towards the center of these states. Attracting state conditions are illustrated in Figure 7A which shows a schema of different kind of convergent trajectories in the high-dimensional state spaces. Figure 7B shows within the 3-dimensional PCA projections some *empirical* examples of such trajectories which either cycle within or return to the task-epoch-specific population states. Figure 7C precisely quantifies, both for the single-trial data sets (red bars, left y-axis) and for the prediction-sets of trials in the multiple-trial data (blue bars, right y-axis), the fraction of trajectories which escaped again from the task-epoch specific clusters without returning to them within the given period (i.e. trajectories which are not of the kind “a” or “b” in Figure 7A). For *O*≈3–5, consistently across all task epochs this was only the case for ∼15% of the trajectories (across all 3 animals) when escape behavior was determined in the prediction trials while event boundaries were those defined in the non-overlapping reference set of trials, as shown in Figure 7C (blue bars, right y-axis; and ∼8% of the escaped trajectories when assessed within the reference set of trials, see red bars, left y-axis). Thus, these results further support the hypothesis that the task-epoch clusters constitute regions of convergence with >80% of trajectories returning to these states or bound within them. In summary, the quantitative analysis of trajectory flows in the optimal state spaces seems to confirm that different cognitively defined task epochs of the present memory-based decision making task act as high probability regions of convergence.

## Discussion

According to many neuro-computational theories, cognitive processes in the brain are implemented through the system's dynamical properties, i.e. the movement of neural trajectories among different attracting states that represent the contents of cognition (e.g. [2], [6], [34]–[36]). A number of previous experimental observations have therefore suggested the existence of attractor-like behavior in the nervous system, or were at least interpreted this way: Many of these studies dealt with forms of persistent [52], [53] or reoccurring spatio-temporal activity patterns [54] as they may be relevant to computational demands in working memory, e.g. temporary active maintenance of stimulus information required in a forthcoming choice situation [2], [5]. Other studies tried to establish direct links between neural attracting behavior and sensory or environmental representations [16], [20], [23]. With the recent progress in multiple single-unit recordings there has also been a rise in the application of advanced techniques from multivariate statistics and machine learning for reconstructing properties of the neural system dynamics or identifying re-occurring patterns, including different dimensionality reduction [16], [33], [55]–[57], pattern classification [33], [55], [58], and time series analysis approaches [18], [19], [59]. However, most of the previous experimental studies inferred attracting dynamics indirectly from, e.g., the property that neural activity after some time settled into one of several discrete states (e.g. [23]). In contrast, a more direct demonstration of a convergent flow of neural trajectories as a defining property of attractor-like structures has been, to our knowledge, mostly lacking so far. This may at least partly be attributed to the methodological difficulties associated with revealing the flow of trajectories directly in the experimentally accessed low-dimensional subspaces, i.e. the spaces spanned by the spiking activities of the set of recorded neurons (cf. Figure 1).

Here we therefore combined well-established approaches from nonlinear dynamics [28], [29] and statistical learning theory [27], [45], [46] for expanding spaces to a sufficiently high dimensionality such that the flow of trajectories becomes resolved. Since the expanded *O^th^*-order embedding space can have very high dimensionality (for instance, for *O* = 5 and *n* = 30 neurons the dimensionality would be on the order of 10^6^), specialized and strongly regularized algorithms (*kernel*-methods) were necessary to perform the relevant computations in these spaces [45]. However, in the present study this was done solely for computational tractability and numerical stability: The kernel function employed here is mathematically equivalent to vector products in the high-dimensional expanded spaces (see Materials and Methods), and hence does not change the nature of any of the results or arguments. We furthermore emphasize that kernel algorithms designed for very high-dimensional systems [26] are well-benchmarked techniques developed during the last decade [44], as are the delay embedding [38] and multinomial basis expansion [45] procedures employed here. All of these methods have been extensively tested with both simulated and real data in many areas of science [26], [44]–[46]. For instance, in functional neuroimaging the usage of kernel methods and high-dimensional classifiers becomes more and more of a routine now (e.g. [60]–[63]). The particular combination of these techniques for their application to electrophysiological data, on the other hand, to our knowledge presents a novel aspect of this work.

Using those approaches, we found that by augmenting the space with dimensions defined as products of neural firing rates, population interaction patterns belonging to distinct, cognitively defined task epochs were maximally separated and predictive of neural-behavioral state associations on future trials (cf. Figures 3, 4 and 7). More importantly, a consistent flow of neural trajectories and their convergence to task-epoch-specific ensemble states became apparent that was not obvious in the lower-dimensional embeddings of neural activity. Thus, the present memory-based decision making task seems to involve different (semi-)attracting states (in a statistical, probabilistic sense) among which neural activity may travel to implement task-related cognitive processes. These states had a cognitive interpretation as they were specific to particular task epochs. The organization of neural activity into different attracting states was furthermore related to behavioral performance: In animals exhibiting a high number of behavioral errors this structure was significantly degraded (Figure 5; see also [33]), perhaps reflecting a general “flattening of attractor basins” associated with diminished memory- and choice-related functions [64]. Therefore our results seem to support long-standing computational theories about the neural implementation of cognitive functions [20], [35], [52]–[54].

### Implications of higher-order neural activity interactions

We observed that unfolding of trajectories and separation of task-epoch clusters became stable across trials when higher-order activity products were taken into account, but did not improve further when moving to arbitrarily high expansion orders. This, in other words, seems to imply that considering the joint activity constellations of a couple of neurons will still add information about the neural dynamics not easily or directly available from single unit activities, while still higher-order interactions may not be relevant: For sub-optimal state spaces the clustering into task-epoch-specific patterns was either unclear (*O* = 1) or had no predictive power across trials (*O*>6; cf. Figure 3). Note, however, that higher-order activity products are used here mainly as a statistical tool for disentangling trajectory flows and not for assessing the cognitive relevance of neural correlations. Thus, we cannot conclusively rule out, for instance, that adding many more neurons and data points to the state spaces than were available in the present study would shift the optimal expansion dimensionality to different orders. The specific value for the optimal expansion order obtained here may just reflect the well-known (in statistics; e.g. [46]) “bias-variance tradeoff” for our data set (in the sense of yielding low generalization errors, i.e. without over-fitting the data).

Nevertheless it is still remarkable that for all the different types of data sets studied here (multiple-trials vs. many animals), different numbers of recorded units, and different numbers of trials (and hence data points) a similar order of activity interactions appeared to be optimal. Similarly, the control studies reported in Figure 4 suggest that sample size effects cannot completely account for the specific optimality value obtained here. Indeed, a recent study, performed in visual cortex, revealed the importance of higher-order correlations in local neural ensembles like recorded here, while only second-order correlations seemed to be the relevant for information transmission across larger cortical distances [65]. The importance of higher-order correlations among neurons for information processing has also been stressed by many previous authors [43], [66], [67], e.g. by relating multiple-spike coincidence statistics to significant behavioral events [40], [42], [68], or by computing the information gained from correlations while decoding the current stimulus from the neural activity [69]. Some research had suggested that higher than second order correlations are redundant, at least in some preparations like the retina which may strongly differ in their structural and computational properties from the neocortex [67]. On the other hand, most recently it was suggested that some of the low bounds found in earlier studies may be an artifact of the limited number of experimentally accessed units [69]. Finally, studies in somatosensory cortex also found similar bounds on the maximum order of perceptually relevant neural activity interactions as suggested here [66].

### Attracting states in neural computation

Within the optimal order expansion spaces, the stable and attracting nature of the task-epoch-specific states became apparent (cf. Figure 7): The neural dynamics progressively slows down as trajectories approach the cluster centers (Figure 6) and the majority of trajectories cycles within or returns towards these states (Figure 7), indicating that there should be bounded regions of the neural state space which capture and contain neural trajectories. Just like in most previous studies indicating attractor-like dynamics (e.g., [16], [20], [23]), we cannot rule out, however, that these states are stimulus-driven, i.e. become attracting states only under the influence of certain (sensory or motor) stimulus conditions, rather than being a property of the intrinsic (autonomous) dynamics. For instance, in Wills et al. [20] or in Niessing and Friedrich [23] the different “categorical” steady state population responses which reflect attracting dynamics are observed for different types of external stimuli (spatial layout of a maze in the first and olfactory composite stimuli in the second case). Likewise, in our case specific spatial, motor, olfactory, or visual properties may be associated with the choice and reward periods.

There are three observations, however, which make it less likely that only external factors account for establishing different attracting states: First, also the delay period where the animals are confined to one arm of the maze and lights are switched off approximately acts as an attracting set of the dynamics, just like the other task epochs (Figures 3B and 5B). Second, the training and test epoch choice periods act as separate attracting states although they should share all sensory and motor features, but differ only in their memory requirements. Third, task-epoch specific states break down if the animals commit a lot of behavioral mistakes in the test period, yet one would assume that they experience similar sensory input and perform similar movements at each choice point. Thus, there must be some internal component in the generation of task-epoch specific states.

Nevertheless, true attractor states as mathematically defined (e.g. [70]) may be unlikely to exist in such an extremely non-stationary and high-dimensional complex system like the neocortex – rather, it seems more likely that neural information processing proceeds by stochastically itinerating among “semi-attracting” states which, for instance, may attract trajectories along most dimensions yet allow them to escape again along others [71]. This idea underlies many more recent conceptualizations of neural information processing (e.g. [35], [72]), and has also been advanced as a theoretical explanation of experimental results on sensory processing in locusts [16], [73]. For instance, a specific population activity pattern may be temporarily stable until some slow negative feedback mechanism has build up sufficiently to inhibit this currently active configuration [74], or until noise has driven the system out of this state again, i.e. until a stochastic transition between states has occurred [24], [25], [75]. It will be very difficult or even impossible to experimentally prove in such a high-dimensional and almost never stationary system under constant bombardment from external sources that any neural activity configuration is formally an attractor. Moreover, whether physiological phenomena as the ones reported here really match formal definitions of attracting states may be largely irrelevant from a computational perspective [35]. Rather, neural objects with semi-attracting properties as shown here could serve equally well (or even better, e.g. with regards to sequence processing) in most computational ideas about cognitive processing.

Does the high expansion order needed to fully reveal the converging dynamics of neural trajectories imply that the attracting states are very high-dimensional? Not necessarily: The key point of the delay embedding is to add more dimensions which are *informative* about the dynamics; many of the single-unit firing rate dimensions may be non-informative, i.e. may not contribute much to disentangling trajectories [28], and thus in principle could be omitted. The multinomial expansion on the other hand primarily serves to optimally pull apart *noisy* trajectories [45]. In a purely deterministic, noise-free system these dimensions would not be needed either to reveal the attractor. Indeed, the fact that convergent properties of the dynamics could be reasonably well evaluated in the 3-dimensional projections obtained by kernel-PCA suggests that the attracting states may in fact live in much lower dimensional subspaces [57]; which however were only fully revealed by properly expanding the space first before reducing it to the most informative dimensions by using kernel-PCA [76].

Finally, we stress that methods like the ones introduced here are widely applicable to almost any multivariate neural time series, including those obtained from various optical or functional imaging techniques, EEG, MEG [60], [63] or electrochemical techniques generating spatio-temporal time series. Thus, they may allow to address a number of previously unanswered questions about neural dynamics in many fields that require a proper unfolding and detailed resolution of trajectories not aided by across-trial averaging. Such techniques may also aid the discovery of common dynamical phenomena across tasks, species, and recording techniques. Here they revealed that ACC networks move among different state space regions, defined by specific population constellations of neural firing rates and their interactions, with a high likelihood of attracting neural trajectories. In this manner ACC networks may parse experience into meaningful task-relevant subcomponents.

## Materials and Methods

### Ethics statement

All animals in this study were treated in accordance with the ethical guidelines set forth by the University of British Columbia and Canadian Council for Animal Care.

### Animals and behavior

Briefly, animals were placed on a reverse light cycle upon arrival and given *ad libitum* access to food for one week. Surgery was then performed and the animals were allowed two weeks of recovery before maze training. For an in depth description of the multi electrode array fabrication and surgical procedures see Lapish et al. [33].

After recovery from surgery, all animals were trained on the delayed spatial win shift run on an eight arm radial maze. Each trial consisted of a training and test phase separated by a one minute delay phase. Prior to the task, the terminal end of all eight arms were baited with a sugar pellet (Research Diets, Inc., New Brunswick, NJ, USA). The training phase commenced by opening four of eight arms. Upon retrieval of the fourth sugar pellet in the training phase, the animal was locked in the last arm visited and the lights were extinguished for the delay. After the delay, the test phase began by allowing access to all eight arms and errors were scored as re-entries into previously visited arms. Upon completion of the trial by retrieving all eight sugar pellets, all arms were closed and the animal was re-confined to the center of the maze. Animals received one trial per day until they made one error or less for two days in a row, and then received a minimum of 10 trials per day. Data sets for the multiple trials analysis were selected from animals that were able to remain vigilant and attend to the task for ∼15 trials as evidenced by uninterrupted foraging.

In order to assess the population dynamic as the cognitive demands of the task vary, the whole time on task was divided into the following six epochs (Figure 2A): reward epochs (dark gray and red dots) during the training or test phases, respectively, correct choice epochs during training and test phases (blue and green, respectively), incorrect arm choice periods (yellow) during the test phase; and the entire delay period (light gray). Reward epochs were defined as the 1 s periods starting 200 ms before the points where the animal's nose reached a food cup during the training and test phase, respectively. Choice epochs were defined as periods starting 1.5 s before the arm choice and finishing 500 ms after it or before the reward period starts (assessed by visual video inspection).

### Electrophysiology

Behavioral data were captured via a video camera (Cohu, Poway, CA, USA), recorded in Noldus Ethovision (Noldus, Leesburg, VA, USA), and exported via voltage in real time as Cartesian coordinates to the Neuralyx recording system and then scored offline. All data was acquired with arrays of 24 single-wire tungsten (diameter = 25 µm, impedance = 150–300 kΏ, California Fine Wire) electrodes implanted into the ACC (Figure S1A). Recordings were sampled at ∼30 kHz, band-pass filtered from 600–6000 Hz, and stored off-line for sorting and analysis. Spike channels were amplified 5,000–10,000 times and thresholds for detection were set to ∼50 µV, which corresponded to >5 times the root mean squared noise amplitude for the system. Spike sorting and classification was performed in Neuralynx Spikesort 3D (Neuralynx, Bozeman, MT, USA). Spike cluster assignments were based upon investigation of numerous principle components of the waveform (), and clusters lacking a well-defined boundary were excluded After classification, unrealistically low ISIs (≤10 ms) were removed as well as neurons with unrealistically high cross-correlations indicating the same neuron may have been captured on two different channels.

### Statistical analysis

An intuitive introduction to our statistical methodology was provided at the beginning of the Results section, while most of the mathematical details can be found in the Supplementary Material.

Spike-trains from the *n* simultaneously recorded units were convolved with Gaussian functions to obtain statistically reliable estimates of spike densities from single trials (checking the range σ = 5–200 ms, see Figure S3 for values from 5–50 ms), normalized to the length of the whole trial (to yield a true probability density) and then summed and binned at 200 ms (approximately the inverse of the average single unit firing rate). Single unit spike densities were then combined into n-dimensional population vectors with components ν*_i_*(*t*) for each unit *i* (e.g. [33], [59] as a function of time bin *t*. Small bin sizes (<50 ms) produce extremely sparse ν*_i_*(*t*) series which became computationally prohibitive for the exact algorithm described below, and numerical approximations were required [45] Units for which 〈ν*_i_*〉 <2% of the most responsive unit were excluded.

For the across-trial analysis, three different datasets consisting of 15 trials recorded on the same day were obtained from 3 animals. For each animal, only trials with ≥20 responsive units (see criterion above) were selected (10 trials from animal #1, 16 trials from animal #2, and 8 trials from animal #3). The first set of trials obeying above criteria constituted the reference set (trials 1–5 for animal #1, trials 1–8 for animal #2, and trials 1–4 for animal #3), while the last set of trials in the sequence formed the prediction set selected such that it had no overlap with the reference set. Furthermore, for each task-epoch, time series from the reference and prediction sets were constrained to have about the same length (number of vectors). For each of these two (reference and prediction) data sets, for each animal firing-rate vectors were then concatenated across trials to yield the two data matrices which entered into the analysis described further below. From our previous study [33] where animals were run only on a single trial after reaching criterion, two separate data sets from six animals were constructed from 8 trials performing with less than two errors (“good performers”) and 8 trials with over four errors (“bad performers”). Neurons from different networks were ordered according to their mean firing-rate, while low-responsive units were excluded as in the multiple-trial dataset.

For standard parametric testing, statistical test details can be found in the corresponding figure captions. For testing attracting properties of the task-epoch sets, nonparametric tests were used based on conservatively designed bootstrap data (100 replications used for one-sided comparisons at p = 0.01) as explained in the corresponding text sections and in Figure S2. For the control analyses shown in Figure 4, original task epochs were artificially augmented 5–20 times (generating ∼10^4^ data points) and decimated by a factor of 0.8-0.6. This process did not significantly alter the original distributions, auto- and cross-correlations for all units.

### State space construction

An instantaneous population firing rate vector in MSUA space, obtained by convolution of the spike trains with Gaussian functions as described above, is given by 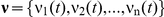 [33]. For univariate time series **ν**(*t*), delay embeddings are usually constructed by forming vectors 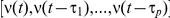 from temporally delayed values with delays (lags) τ*_i_*. These are typically chosen to correspond to *p* successive minima of the autocorrelation function (or mutual information) where *p* would be high enough to unfold (pull apart) trajectories within this delay-coordinate space [38], [77]. In general, the reconstructed spaces should have dimensionality *p* = 2×*D*+1, where *D* is the attractor dimension [28]. Similar ideas can be applied to multivariate systems [29]. The attractor dimension is often estimated via the correlation dimension, which, however, will not provide sensible answers in sparse high-dimensional spaces as the ones examined here (see [29]; Figure S4 and Text S2). Moreover, the use of large delay coordinate maps would result in an extensive loss of data and hence poor statistics. Therefore, additional non-delay variables are sought to effectively disentangle noisy trajectories.

The first step in our approach is to construct a reduced multivariate delay-coordinate map which should simply ensure that trajectories do not significantly cross each other. This auxiliary DC-MSUA space, defined by vectors 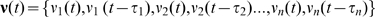, contains only a single lag for each unit optimized to be the first minimum of the average cross-correlation between pairs of units' firing-rates. The resulting lags ranged from just one time bin to <5% of the task-phase length. Note that the main purpose of these lagged variables is to add axes to the space which contain information about the system dynamics not captured by the current state of the firing rate variables, therefore the choice of lags such as to minimize cross-correlations. In fact, the use of more than one delay per unit did not improve across-trial predictions (data not shown).

After this step, differences between trajectories were further amplified by combining these variables into new functional forms, in accordance with ideas from statistical learning theory [27]. As we were specifically interested in functional forms with a biological meaning, this was done by adding higher-order products of the units' firing rates as new coordinates to the neural state space. The *o^th^*-order interaction of *n*-units omitting lags for notational convenience, is defined by
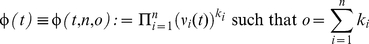
(1)By construction of the smoothed firing rate vector (see above) each axis ϕ(*t*) is the net sum of probabilities of *o* multiple spikes independently occurring across *n* neurons (e.g. [78]). For the frequent case that a single spike is contained within a single bin, and for small smoothing windows σ, ϕ(*t*) tends to represent a pattern of multiple spike-co-occurrences (a “poly-synchronous” pattern [79]). Now, the *O* ^th^-order delay-interactions coordinate map consists of *all o* ^th^-order firing-rate products with *o = 1…O*. Vectors in this high-dimensional space will be denoted by **Φ**(*t*). For instance, a vector in the space corresponding to *O* = 2 is defined by
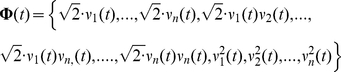
(2)The dimensionality *p*(*O*) of such a space is typically *p*∼10^5^–10^9^, much larger than the number of task-epoch vectors which is on the order of ∼10^3^. Note that this approach sharply contrasts with other methods where the MSUA space dimensionality is instead further reduced by exploiting correlations among units (e.g. [14], [15], [80]). As was noted further above, the delay-coordinate map suffices to remove overlap between trajectories in an ideal, purely deterministic system. On the other hand, the multinomial basis expansion defined above helps to achieve an optimal separation in a statistical learning sense when dealing with highly noisy systems (e.g. Figure 3).

Explicit computations in such extremely high-dimensional spaces are associated with numerical and computational problems which can be solved by the so-called “kernel trick” [45]. In this context a kernel is a function which represents a vector product in a high-dimensional space without explicitly computing the dot product of the vectors. Here, for any two high-dimensional vectors **Φ**(*t*) from the expanded *O^th^*-order space occurring at times *t_a_* and *t_b_*, respectively, the kernel function is given by



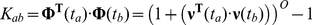
(3)Thus, the function on the right hand side operating on the low-dimensional firing rate vectors **ν**(*t*) is mathematically equivalent to (and uniquely defined for) a dot product between vectors **Φ**(*t*) from the much higher-dimensional *O^th^*-order space [45]. See Text S1 and [81] for further motivation for the use of this kernel.

### State space analysis

Within the mathematical framework of kernel algorithms, high-dimensional covariance matrices are replaced by kernel matrices in the reformulation of classical statistical procedures like PCA or FDA. Kernel matrices were computed for each possible pair of task epochs (such that *t*_a_ and *t*_b_ in Equation 4 may correspond to two different time points of the same epoch, or to time points from two different epochs), and then used to build a classifier using Fisher's discriminant (FD) criterion. FD analysis works by maximizing the difference between task-epoch means while minimizing within-task-epoch (co-) variances, i.e., by finding the direction **Ω** of the high-dimensional *O^th^*-order space along which the overlap between two task-epoch distributions is minimized [45], [50]. Since in the expanded spaces the number of dimensions (variables) *d* is extremely high, in fact much higher than the number of observations *m*, means and covariance matrices cannot be explicitly computed, as stated above, and thus for the FD analysis all computations on high-dimensional vectors are reformulated in terms of a kernel matrix *K* of much smaller dimensionality (equal to *m^2^*<<*d* ^2^; see Text S1). By usage of the kernel matrix *K*, the projections *x*(*t*_i_) of high-dimensional vectors **Φ**(*t*_i_) onto the optimally discriminating direction **Ω** are obtained by
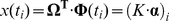
(4)where the *m* elements of the vector **α** are derived as e.g. explained in Schölkopf and Smola, [45] and in Text S1. Since the projected values *x*(*t*_i_) on the most discriminating axis represent linear combinations of up to 10^9^ random variables (one variable per dimension), the projected data will be approximately normally distributed according to the central limit theorem (e.g. [44]). Hence, building on this assumption of approximate normality, a Bayes-optimal classifier (the one with theoretically best performance) can be defined on this most-discriminating axis (where equal priors were used here for not biasing the results according to the lengths of the sampled task epochs). From this, classification (separation) errors (SE; cf. Figure 5), likelihoods *p*(**ν**(*t*)|*C*) of classification into task-epoch *C*, posterior probabilities *P*(*C|* **ν**(*t*)) (using Bayes criterion), and 99% confidence intervals are straightforward to obtain. The (discretized) Kullbach-Leibler divergence [e.g. 44] was computed as a measure of the distance between these Gaussian posterior distributions corresponding to any two tasks epochs *C_1_* and *C_2_*. It was estimated for each *O^th^* order expansion (Figure 5C) and is given by
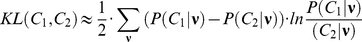
(5)The utilization of normal probability theory represents a fundamental advantage over other approaches specialized for high-dimensional spaces (e.g. support-vector-based classifiers [27]) which may have similar classification performance [45] but do not easily permit other aspects of the present statistical analysis.

For the across-trial analyses, optimal directions **Ω** for each task epoch pair were obtained using exclusively the first set of (reference) trials, **Φ** ^r*ef*^. This direction was then fixed for computing the projections *x_predic_*(*t*_i_) of vectors **Φ** *^predic^*(*t*_i_) from the prediction set onto **Ω** to yield the predicted SE (SE_predic_):
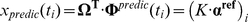
(6)where the vector **α^ref^** is the one obtained from the reference set and *K* represents projections of prediction set vectors into the reference space. A brief summary of these algorithms can be found in Text S1 [45].

A regularization penalty was furthermore added to the kernel matrices to ensure a low *generalization error* (loosely speaking, a regularization factor automatically constrains the number of free parameters to reduce out-of-sample prediction errors; e.g. [27], [45]). This regularization was optimized such that SE_predic_ was minimal for animal #1 and then it was fixed for all other analyses (because of this regularization, for instance, *in-sample* SE never decreases to zero for the expanded spaces in Figures 3A and 5A). Prediction errors were found to be invariant for large enough values of this regularization penalty (as demonstrated in Figure S3). The robustness of the present approach with regards to different basis functions used in the expansion (and thus different definitions of the kernel) is also discussed in Figure S3. Finally, we also investigated how unsupervised clustering approaches perform on the DC-MSUA spaces, and noticed that they reliably pick up only the delay vs. training/test phase differences in this lower-dimensional representation (see Figure S5 for an example).

Kernel-FDA [50] and kernel-PCA [47] were used to obtain three-dimensional visualizations for each high-dimensional task-epoch state. Three-dimensional projections were also used for determining velocity vectors (cf. Figure 6), as these cannot be efficiently computed in high dimensions (a problem running under the label “curse of dimensionality”; e.g. [44]). Kernel-PCA proceeds in much the same way as ordinary PCA, except that – like kernel-FDA – it works on the kernel matrices defined above instead of directly on the high-dimensional covariance matrices (see brief summary in Text S1). Thus, the three orthogonal dimensions capturing the largest amount of data variance in the high-dimensional spaces were obtained. Additional discussion about the adequacy of these three-dimensional velocity vectors as obtained by kernel-PCA can be found in Figure S4 and in Text S2. Finally, note that, apart from the convergence analysis shown in Figure 6, these three-dimensional reductions served only for the purpose of visualization, while all statistical analyses were performed on the full high-dimensional spaces (see Figure 7C).

Analysis software was implemented in MatLab (Mathworks Inc., MA, USA) and is freely available in http://www.bccn-heidelberg-mannheim.de under the terms of the general public license (http://www.gnu.org/licenses/).

## Supporting Information

Figure S1 Multiple single-unit recordings from ACC in a memory-guided decision making task. **A.** Electrode location. All brains were sectioned and electrode placement confirmed. Gray circles delineate the boundary within which electrodes were placed and were confirmed to be in the Anterior Cingulate Cortex (ACC). Cortical map is adapted from Paxinos and Watson [82]. **B.** A representative example of a channel containing 3 units. The averaged waveform is shown on top with the color corresponding to the cluster in the map below.
(TIF)

Figure S2 Schema of the bootstrap procedures. **A.** Original data. **B.** Bootstrap series used in Figures 3 and 4 were constructed by randomly shuffling stretches of the time series that retained entire trajectories form a given task epoch such that each replication preserved all temporal autocorrelations up to the length of the relevant task epoch.
(TIF)

Figure S3 Robustness of the state space reconstruction approach to different parameter settings. **A.** Misclassification error, SE, for different standard deviations (σ) of the Gaussian smoothing function used for constructing the firing-rate vectors. Blue and red lines show SE for low- and high-performance trials, respectively. Results are averages across all task-epoch pair comparisons (error bars = SEM). Results are largely the same for 5<σ<200 ms. **B.** SE for different settings of the regularization parameter of the kernel matrix which penalizes the number of state space dimensions [45], [83]. Regularization (η) is expressed as % of the mean value of the kernel matrix (see Text S1). For η<1%, SE approaches zero (p>0.5) for sufficiently high *O*, and the discrimination between low- and high-performance trials disappears, while for larger values (∼1–40%), discrimination between behavioral performance groups is retained. **C.** SE_predic_ in the optimum expansion spaces as a function of the regularization parameter. Very low penalties (η≤1%) are associated with larger SE_predic_ while for η>1% mean SE_predic_ does not change anymore (n = 3 animals). This result indicates that the very low “naïve” SEs obtained in graph B for η<1% are purely due to “overfitting” [44], [45] and therefore are not informative. Beyond this lower limit for η, results of this study are largely independent of this regularization parameter. **D.** SE_predic_ for an optimal *O^th^*-order space which, however, contains only interactions of *O^th^*-order, and not those of order *o*<*O* (black bars). In contrast to the *O^th^*-order space used in this work (white bars), this space is not functionally meaningful in the sense that high-order spike correlations across neurons necessarily imply lower order ones which are not present in this space. However, one would expect that SE_predic_ within such a space remains unaltered because the change in dimensionality is negligible (e.g., for *O* = 3 the dimensionality would decrease only by ∼8% by neglecting lower order interactions, while instead the dimensionality increases by ∼900% when going from *O* = 3^rd^ to *O* = 4^th^ order). Surprisingly, however, SE_predic_ deteriorates for these “non-meaningful” spaces despite their similar dimensionality, indicating that the optimum full *O^th^*-order embedding space is also the functionally most relevant one.
(TIF)

Figure S4 Assessment of the validity of the three-dimensional kernel-PCA projections for representing task-epoch-specific dynamics within the optimal full *O^th^*-order space. See discussion of this Figure in Text S2. **A.** Correlation dimension (*d_2_*) of task-epoch specific sets in the three-dimensional space obtained from a *5^th^*-order expansion during high-performance trials. *d_2_* is defined as the slope of *log S*(ε)- *log*(ε) in the limit of an infinite number of samples and ε→0, where *S*(ε) is the fraction of data points falling into spheres of radius ε centered on each of the data points in turn (termed *correlation sum*; [29]). The inset shows the mean Takens maximum likelihood estimator of *d_2_* [84], which turns out to be smaller than one. According to the fractal delay-coordinate embedding theorem [28], the minimum required dimensionality of a proper low-dimensional embedding for each of the task-epoch-specific putative *attractors* is therefore 3 (i.e. 2 *d_2_*+1). Thus, these three-dimensional visualizations provide reliable representations of task-epoch trajectories. **B.** *Time-Space separation plots* [85] used to estimate the minimum number of temporally consecutive vectors (abscissa), *Δsamples*, which should not be included in the *S*(ε) counts, termed *b_min_*. Since *d_2_* is supposed to be a measure of the spatial geometry of a putative attractor [86], spatial neighborhood relationships purely due to different short-term autocorrelations within trajectories have to be excluded by choosing *b_min_* appropriately. For *b_min_* = 4–29 samples the variation in *S*(ε) across *Δ* *samples* was less than 5% for all ε.
(TIF)

Figure S5 Example of unsupervised hierarchical clustering analysis performed on the DC-MSUA space for animal #1. For the purpose of visualization, 3D-projections obtained by PCA or Multi-Dimensional Scaling are shown. **A.** Optimal unsupervised two-group clustering solution showing the delay phase in gray and the training plus test phases in brown (these were the most distinct classes as revealed by the dendrogram shown in the upper right). Different clustering criteria (centroid, average, median, nearest neighbor, weighted and Ward's) and metrics (Euclidean, various Minkowsky metrics, Mahalanobis, and Pearson correlation) were tried. The “optimal” clustering criterion (Ward's in this case) and optimal metric (Euclidean in this case) were the ones which yielded the lowest percent of misclassified firing-rate vectors (CE) with respect to the experimenter-determined task-phase assignments. The upper graphs show results for trial #3, where only 15.5 (3.0) % of vectors were misclassified on average across task-phases (standard error), while the bottom graph shows results when all training set trials (#1–5) were combined, yielding an average of 30.0 (9.3) % misclassified vectors. These results suggest that within the DC-MSUA spaces unsupervised methods reliably pick up the difference between the delay phase and the other task phases. **B.** Optimal six-group clustering solution. In this case any of the hierarchical clustering methods resulted in an average number of misclassified vectors >30% (mean CE across the six task-epochs is 69.3 (11.7) %). Thus, at least within the low-dimensional DC-MSUA spaces unsupervised methods were not able to reliably detect different task-epochs with predictive power. This of course does not rule out that *kernelized* versions of unsupervised cluster analyses could identify task epochs in the high-dimensional expanded spaces, an issue which can be studied in future extensions of the present work.
(TIF)

Text S1 Brief summary of kernel algorithms.
(DOC)

Text S2 Three-dimensional representations.
(DOC)

Video S1 Kernel-PCA reduction of the expanded space containing higher-order activity products (Figure 1B, right), showing attracting-like orbits for the different task-phases.
(AVI)
