# Supplementary figures and images for: Attracting Dynamics of Frontal Cortex Ensembles during Memory-Guided Decision-Making
Source: PLoS Comput Biol. 2011 May 19;7(5):e1002057. doi: 10.1371/journal.pcbi.1002057 (PMC3098221; doi:10.1371/journal.pcbi.1002057)

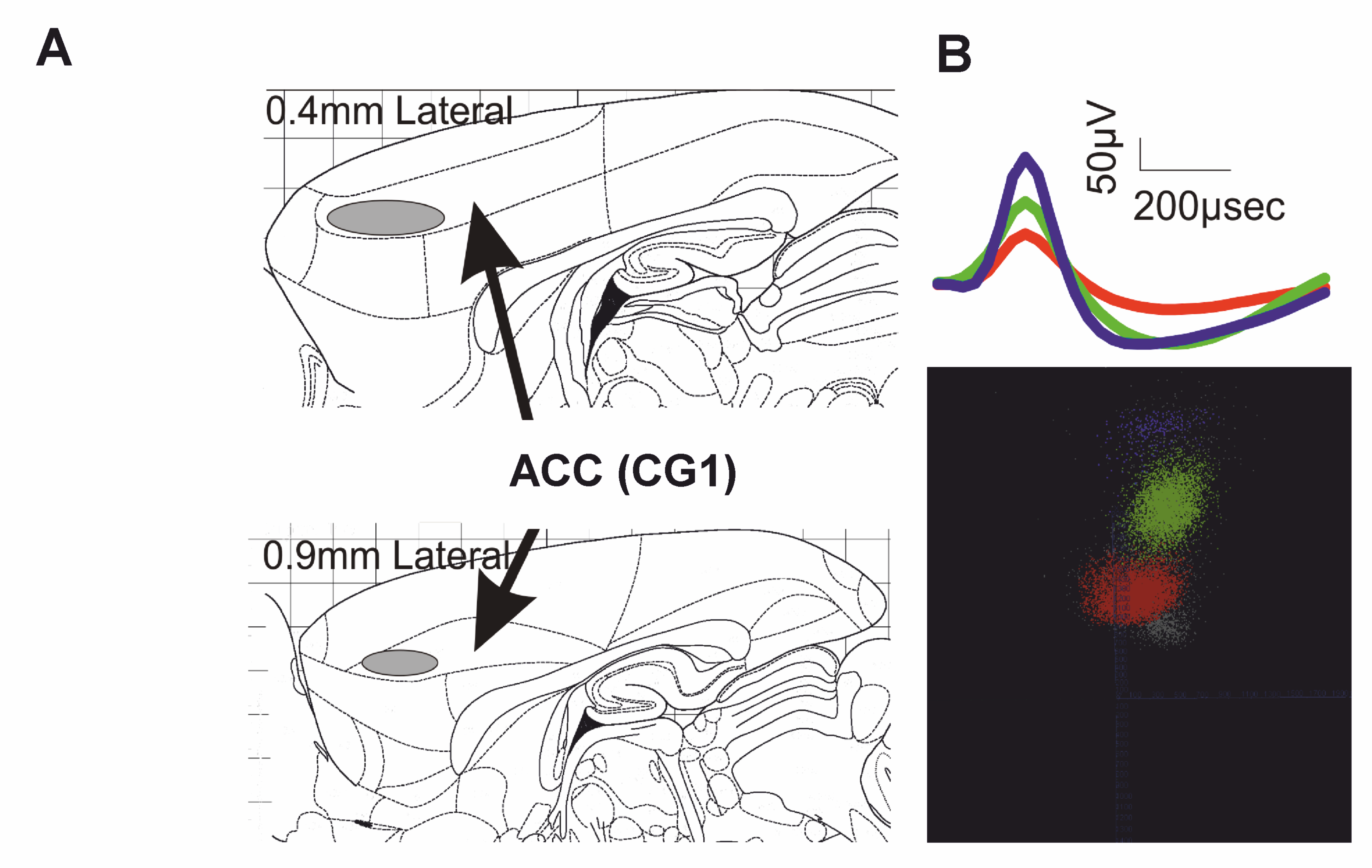

Supplement: Figure S1 — Multiple single-unit recordings from ACC in a memory-guided decision making task. A. Electrode location. All brains were sectioned and electrode placement confirmed. Gray circles delineate the boundary within which electrodes were placed and were confirmed to be in the Anterior Cingulate Cortex (ACC). Cortical map is adapted from Paxinos and Watson [82]. B. A representative example of a channel containing 3 units. The averaged waveform is shown on top with the color corresponding to the cluster in the map below. (TIF) [file pcbi.1002057.s001.tif]

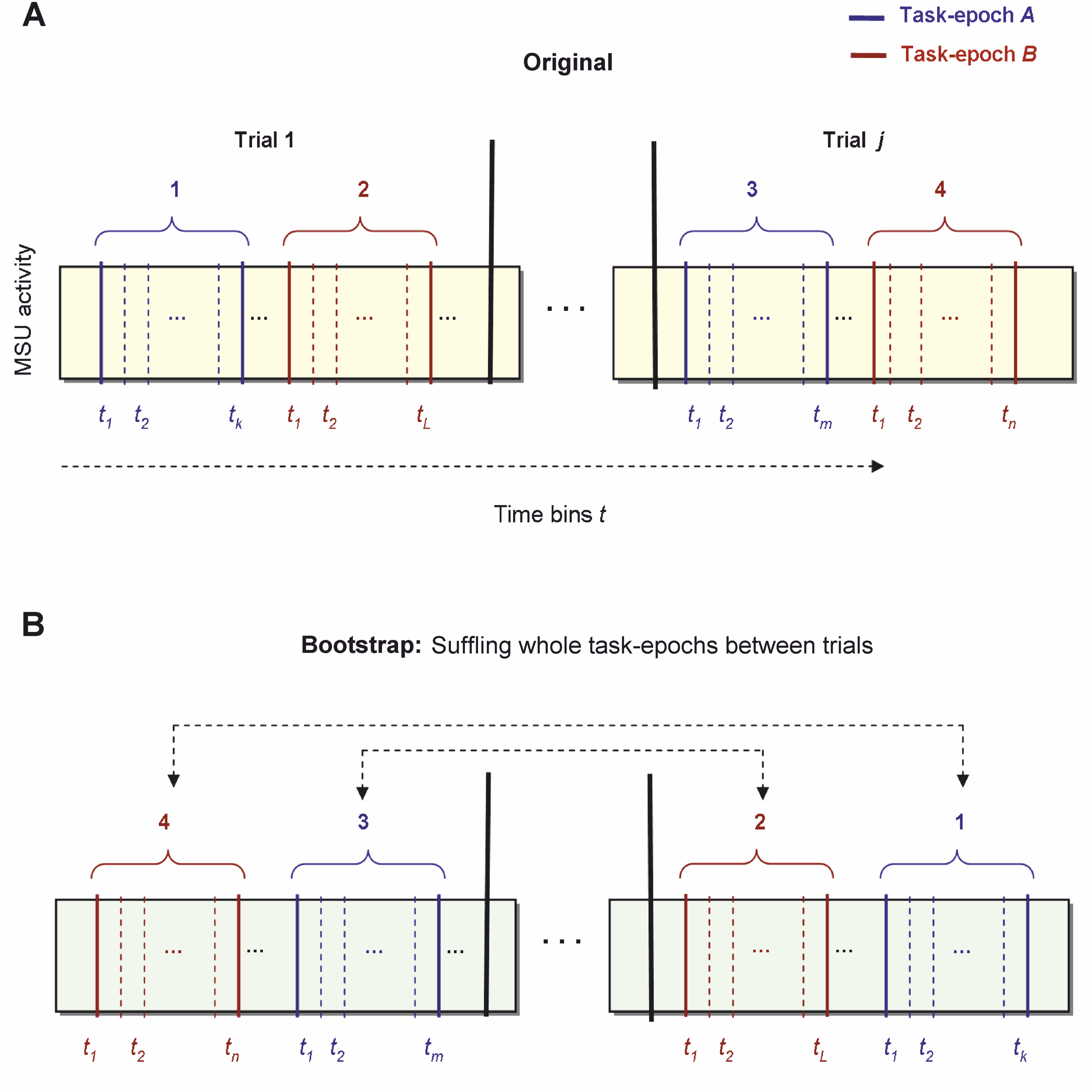

Supplement: Figure S2 — Schema of the bootstrap procedures. A. Original data. B. Bootstrap series used in Figures 3 and 4 were constructed by randomly shuffling stretches of the time series that retained entire trajectories form a given task epoch such that each replication preserved all temporal autocorrelations up to the length of the relevant task epoch. (TIF) [file pcbi.1002057.s002.tif]

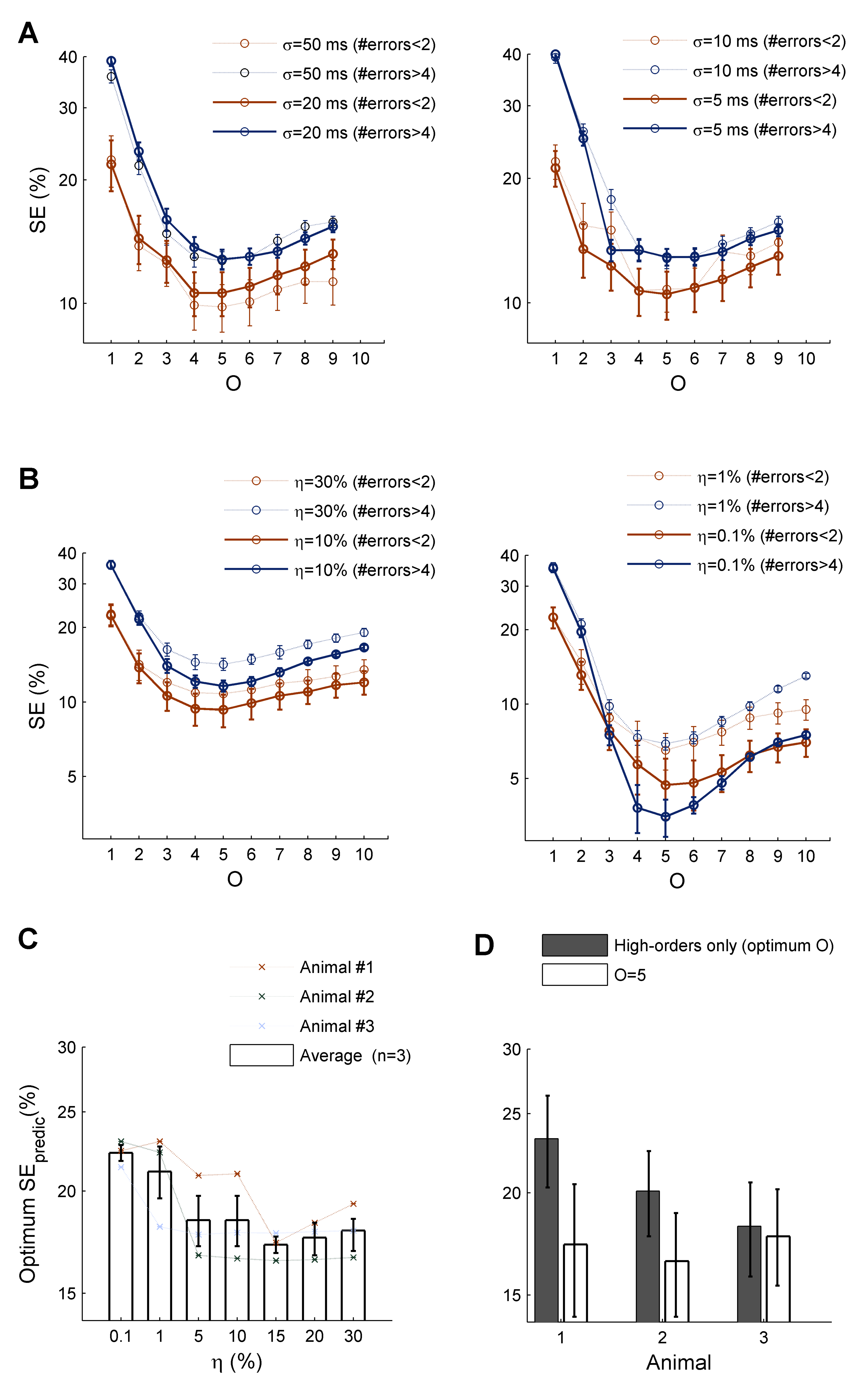

Supplement: Figure S3 — Robustness of the state space reconstruction approach to different parameter settings. A. Misclassification error, SE, for different standard deviations (σ) of the Gaussian smoothing function used for constructing the firing-rate vectors. Blue and red lines show SE for low- and high-performance trials, respectively. Results are averages across all task-epoch pair comparisons (error bars = SEM). Results are largely the same for 5<σ<200 ms. B. SE for different settings of the regularization parameter of the kernel matrix which penalizes the number of state space dimensions [45], [83]. Regularization (η) is expressed as % of the mean value of the kernel matrix (see Text S1). For η<1%, SE approaches zero (p>0.5) for sufficiently high O, and the discrimination between low- and high-performance trials disappears, while for larger values (∼1–40%), discrimination between behavioral performance groups is retained. C. SEpredic in the optimum expansion spaces as a function of the regularization parameter. Very low penalties (η≤1%) are associated with larger SEpredic while for η>1% mean SEpredic does not change anymore (n = 3 animals). This result indicates that the very low “naïve” SEs obtained in graph B for η<1% are purely due to “overfitting” [44], [45] and therefore are not informative. Beyond this lower limit for η, results of this study are largely independent of this regularization parameter. D. SEpredic for an optimal Oth-order space which, however, contains only interactions of Oth-order, and not those of order o<O (black bars). In contrast to the Oth-order space used in this work (white bars), this space is not functionally meaningful in the sense that high-order spike correlations across neurons necessarily imply lower order ones which are not present in this space. However, one would expect that SEpredic within such a space remains unaltered because the change in dimensionality is negligible (e.g., for O = 3 the dimensionality would decrease only by ∼8% by neglecti [file pcbi.1002057.s003.tif]

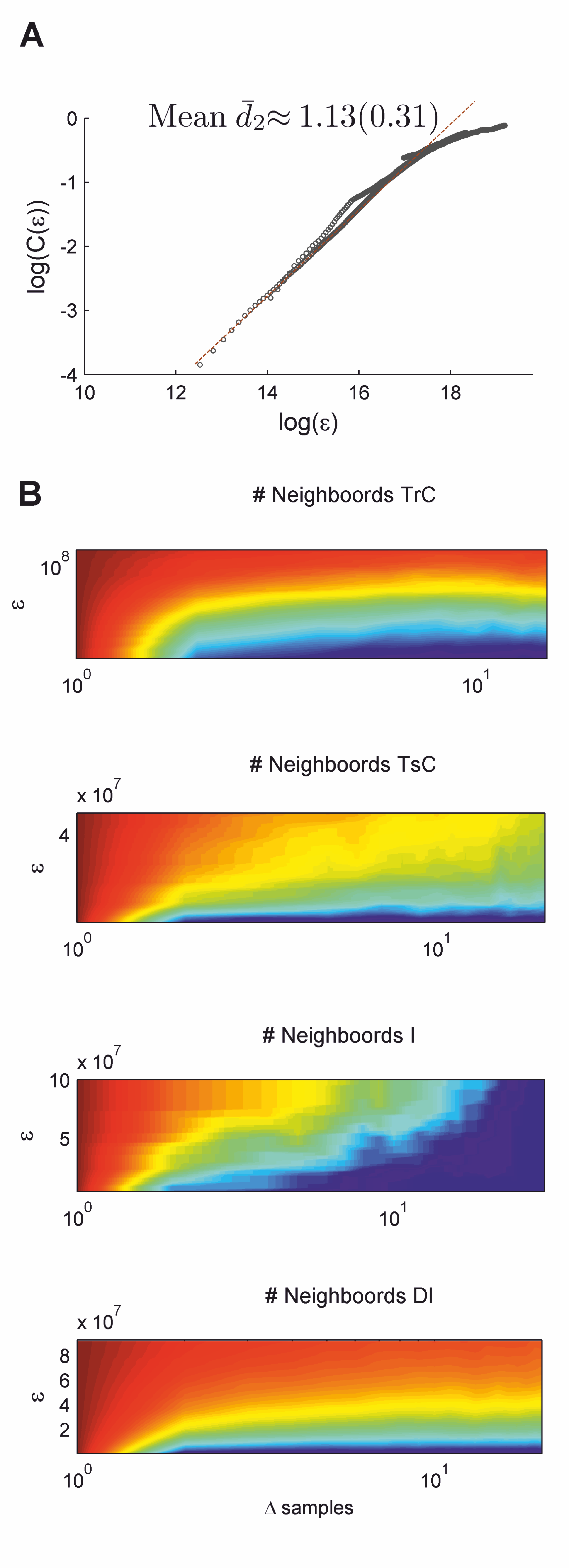

Supplement: Figure S4 — Assessment of the validity of the three-dimensional kernel-PCA projections for representing task-epoch-specific dynamics within the optimal full Oth-order space. See discussion of this Figure in Text S2. A. Correlation dimension (d2) of task-epoch specific sets in the three-dimensional space obtained from a 5th-order expansion during high-performance trials. d2 is defined as the slope of log S(ε)- log(ε) in the limit of an infinite number of samples and ε→0, where S(ε) is the fraction of data points falling into spheres of radius ε centered on each of the data points in turn (termed correlation sum; [29]). The inset shows the mean Takens maximum likelihood estimator of d2 [84], which turns out to be smaller than one. According to the fractal delay-coordinate embedding theorem [28], the minimum required dimensionality of a proper low-dimensional embedding for each of the task-epoch-specific putative attractors is therefore 3 (i.e. 2 d2+1). Thus, these three-dimensional visualizations provide reliable representations of task-epoch trajectories. B. Time-Space separation plots [85] used to estimate the minimum number of temporally consecutive vectors (abscissa), Δsamples, which should not be included in the S(ε) counts, termed bmin. Since d2 is supposed to be a measure of the spatial geometry of a putative attractor [86], spatial neighborhood relationships purely due to different short-term autocorrelations within trajectories have to be excluded by choosing bmin appropriately. For bmin = 4–29 samples the variation in S(ε) across Δ samples was less than 5% for all ε. (TIF) [file pcbi.1002057.s004.tif]

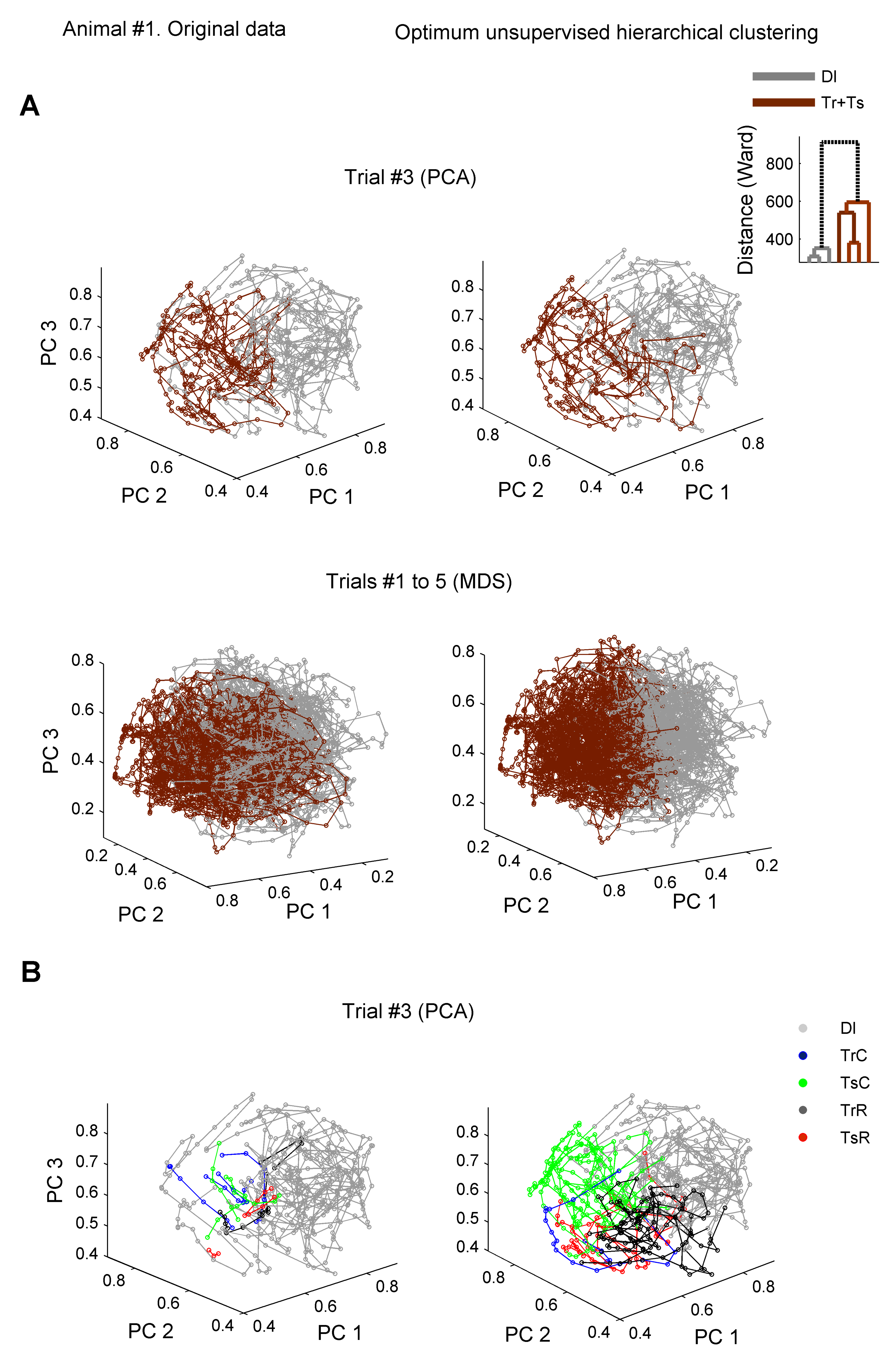

Supplement: Figure S5 — Example of unsupervised hierarchical clustering analysis performed on the DC-MSUA space for animal #1. For the purpose of visualization, 3D-projections obtained by PCA or Multi-Dimensional Scaling are shown. A. Optimal unsupervised two-group clustering solution showing the delay phase in gray and the training plus test phases in brown (these were the most distinct classes as revealed by the dendrogram shown in the upper right). Different clustering criteria (centroid, average, median, nearest neighbor, weighted and Ward's) and metrics (Euclidean, various Minkowsky metrics, Mahalanobis, and Pearson correlation) were tried. The “optimal” clustering criterion (Ward's in this case) and optimal metric (Euclidean in this case) were the ones which yielded the lowest percent of misclassified firing-rate vectors (CE) with respect to the experimenter-determined task-phase assignments. The upper graphs show results for trial #3, where only 15.5 (3.0) % of vectors were misclassified on average across task-phases (standard error), while the bottom graph shows results when all training set trials (#1–5) were combined, yielding an average of 30.0 (9.3) % misclassified vectors. These results suggest that within the DC-MSUA spaces unsupervised methods reliably pick up the difference between the delay phase and the other task phases. B. Optimal six-group clustering solution. In this case any of the hierarchical clustering methods resulted in an average number of misclassified vectors >30% (mean CE across the six task-epochs is 69.3 (11.7) %). Thus, at least within the low-dimensional DC-MSUA spaces unsupervised methods were not able to reliably detect different task-epochs with predictive power. This of course does not rule out that kernelized versions of unsupervised cluster analyses could identify task epochs in the high-dimensional expanded spaces, an issue which can be studied in future extensions of the present work. (TIF) [file pcbi.1002057.s005.tif]
