# Supplementary material for: Attracting Dynamics of Frontal Cortex Ensembles during Memory-Guided Decision-Making
Source: PLoS Comput Biol. 2011 May 19;7(5):e1002057. doi: 10.1371/journal.pcbi.1002057 (PMC3098221; doi:10.1371/journal.pcbi.1002057)
Supplement: Text S1 — Brief summary of kernel algorithms. (DOC) [file pcbi.1002057.s006.doc]

Text S1. Brief summary of kernel algorithms

The present summary is mainly a synthesis from previous publications by Robert-Müller, Schölkopf, Smola and colleagues [45, 47, 50, 76, 81] with the terminology adapted for this work for clarity.

**Kernel function and multinomial expansion.** The kernel function given in Equation 3 (termed an *inhomogeneous polynomial*) is an *admissible* kernel, i.e. it is possible to define a function from the (DC-)MSUA space into H*^D(O)^* where *p*>>*n* (*n*=number of units) such that this kernel is exactly equivalent to a dot product **Φ^T^ Φ** of any two vectors **Φ** in the high dimensional space H*^D^* (this is demonstrated for this precise kernel in [81]). The dimensionality of such a space is the total number of terms within a multimomial expansion. For correctly defining such a space, elements of **Φ** are multiplied by the square root of the multinomial expansion coefficients, e.g. see Equation 2 in Materials and Methods. For instance, for *O*=3 and *n*=2 (and again omitting time lags for simplicity), the dot product is

$$\Phi^{T}(t_{a})\cdot\Phi(t_{b})=$$

$$\sqrt{3}^{2}\cdot\nu_{1}(t_{a})\nu_{1}(t_{b})+\sqrt{3}^{2}\cdot\nu_{2}(t_{a})\nu_{2}(t_{b})+\sqrt{3}^{2}\cdot\nu_{_{1}}^{2}(t_{a})\nu_{1}^{2}(t_{b})+\sqrt{3}^{2}\cdot\nu_{2}^{2}(t_{a})\nu_{2}^{2}(t_{b})$$

$$+\sqrt{6}^{2}\cdot\nu_{1}(t_{a})\nu_{2}(t_{a})\nu_{1}(t_{b})\nu_{2}(t_{b})+\sqrt{3}^{2}\cdot\nu_{1}^{2}(t_{a})\nu_{2}(t_{a})\nu_{1}^{2}(t_{b})\nu_{2}(t_{b})$$

$+\sqrt{3}^{2}\cdot\nu_{2}^{2}(t_{a})\nu_{1}(t_{a})\nu_{2}^{2}(t_{b})\nu_{1}(t_{b})+\nu_{1}^{3}(t_{a})\nu_{1}^{3}(t_{b})+\nu_{2}^{3}(t_{a})\nu_{2}^{3}(t_{b})=\left(1+\nu^{T}(t_{a})\cdot\nu(t_{b})\right)^{3}-1=K_{ab}$.

***Kernelizing* classical statistical tools**. Classical covariance-matrix-based statistical tools can be reformulated in terms of kernel matrices for very high-dimensional spaces. For example, a kernel Fisher Discriminant [50], like an ordinary Fisher discriminant, finds a direction **Ω**ÎH*^D^* (where H*^D^* is a *D*-dimensional *Hilbert* space) in the high-dimensional spaces such as to maximize the distance between two cluster centroids while at the same time minimizing the within-cluster jitter, i.e. maximizes

$J(\Omega)=\frac{\Omega^{T}\cdot M\cdot\Omega}{\Omega^{T}\cdot N\cdot\Omega}$ (S1)

where $M=\left(m_{q}-m_{r}\right)\cdot\left(m_{q}-m_{r}\right)^{T}$, $m_{q}=\left\langle\Phi(t_{q})\right\rangle$ and $m_{r}=\left\langle\Phi(t_{r})\right\rangle$ are the means of task-epochs *q* and *r* having *Q* and *R* vectors, respectively, and *N* is the high-dimensional (i.e., high-order in here) covariance matrix i.e.

$N=\left\langle(\Phi(t_{q})-m_{q})\cdot(\Phi(t_{q})-m_{q})^{T}\right\rangle+\left\langle(\Phi(t_{r})-m_{r})\cdot(\Phi(t_{r})-m_{r})^{T}\right\rangle$.

However, matrices *N* and *M* are *D x D*-dimensional (up to ~10^9^ x 10^9^) and thus the solution to this problem cannot be computed directly. Therefore, in Mika et al. [50] Equation S1 was reformulated in terms of a much smaller kernel function, which amounts to finding **α**ÎÂ*^Q+R^* such that

(S2)

is maximized, where $\tilde{M}$ and $\tilde{N}$ are now of much smaller dimension *Q+R x Q+R* (~10^3^ x 10^3^), i.e. the same dimensionality as for the kernel matrix *K* corresponding to a pair of task-epochs as given in Materials and Methods (see [45], for the exact form of $\tilde{M}$ and $\tilde{N}$). The much reduced dimensionality of this problem enables to obtain the vector **α** which solves (S2) and thus the optimum discriminating direction **Ω** (see Equation S4 below). Further details of this algorithm can be found in Schölkopf and Smola [45]. The maximization is achieved by solving the following eigenvalue problem:

(S3)

where η *K* is a regularization penalty [50] analyzed in Figure S3. Like with conventional linear discriminant analysis, vectors **Φ** of a task-epoch *C* are projected onto the optimal discriminant direction **Ω**ÎH*^p^*. Again this can be done without explicitly representing such high-dimensional vectors by using the kernel matrix:

$\Omega^{T}\cdot\Phi(t_{c})=\sum_{b=1}^{Q+R} \alpha_{b}\cdot K_{bc}=\left(K\cdot\alpha\right)_{c}$ (S4)

Kernel PCA [47] is a reformulation of classical PCA which can be used in cases where the covariance matrix is so extremely high dimensional that its eigenvector decomposition cannot be effectively obtained, like it was the case for FDA introduced above. The principal components are the eigenvectors **γ**Î H*^D(O)^* of a *D* x *D* high-dimensional covariance matrix *N*, i.e.

$N\cdot\gamma=\lambda\cdot\gamma$. (S5)

Kernel PCA circumvents the problem of having to compute these eigenvectors directly for the very high-dimensional matrix *N* by computing the eigenvectors of the kernel matrix instead, i.e. eigenvectors **β** and eigenvalues λ of a (centered) kernel matrix *K* are obtained by solving the problem

$K\cdot\beta=\lambda\cdot\beta$. (S6)

The high-dimensional eigenvectors **γ** can now be expressed as a function of **β** as shown in [45]. Finally, as in conventional PCA, the low-dimensional representation $\Psi(\nu(t))\equiv\left\{\text{Ψ}_{1}(\nu),\text{Ψ}_{2}(\nu),\text{Ψ}_{3}(\nu)\right\}$ of any vector **Φ**(*t*) is constructed by projecting it onto each one of the three main eigenvectors (those associated with the largest variance directions), which is again feasible with the aid of the kernel matrix (cf. Equation 4),

$\Psi(\nu(t))=\gamma^{T}\cdot\Phi(t)=\left(K\cdot\beta\right)_{t}$ (S7)

Further details of this method can be found in [45, 47].
