# Supplementary material for: Attracting Dynamics of Frontal Cortex Ensembles during Memory-Guided Decision-Making
Source: PLoS Comput Biol. 2011 May 19;7(5):e1002057. doi: 10.1371/journal.pcbi.1002057 (PMC3098221; doi:10.1371/journal.pcbi.1002057)
Supplement: Text S2 — Three-dimensional representations. (DOC) [file pcbi.1002057.s007.doc]

Text S2. Three-dimensional representations

This section discusses results shown in Figure S4 and justifies the use of three dimensional velocity vectors for the convergence tests shown in Figure 6. According to the fractal delay-embedding prevalence theorem for noise-free *deterministic* systems [28] a smooth delay-coordinate map is an embedding if its dimensionality is larger than twice the *box-counting* dimension, *d_0_*, of the underlying attractor, where by embedding a space is meant that fully (without loss of information) captures the complete geometry of the *attractor*. In principle an attractor dimension of each putative task-epoch attracting state could be estimated from any delay-coordinate map of sufficiently high dimensionality or, in general, from any large enough set of independent dimensions of the system. However, due to the poor statistics in very high-dimensional spaces (number of data points similar or smaller than number of dimensions) and biases in the distribution of vector distances (‘curse of dimensionality’ e.g. [44]) this becomes unfeasible in the DC-MSUA spaces and of course in the *O^th^-order* spaces considered here. We therefore defined a low-dimensional and non-sparse space in which axes are only weakly correlated like in a delay-coordinate map for each attracting state.

The first requirement was that all axes in the map are weakly correlated among each other. Only the actual value of the most responsive unit and delayed axes of all other units were retained in the delay-coordinate representation. Second, since low-dimensional (and non-sparse) projections **Ψ** obtained by kernel-PCA are by definition de-correlated, the projected attracting state has a dimension that could be estimated from this space of orthogonal axes. An upper bound for *d_0_* is given by the correlation dimension, *d_2_* [86]. Therefore, we estimated the correlation dimensions in the 3-dimensional projections separately for each task-epoch *q* across all trials of the task (with a total of *Q* vectors). The correlation dimension is defined as [29]

$d_{2}(q)=\lim_{\begin{matrix} Q\to\infty\\ \varepsilon\to0 \end{matrix}}\frac{\partial\ln S(\varepsilon,Q)}{\partial\ln\varepsilon}$ where the correlation sum *S* is given by

$S(\varepsilon,Q,b_{\min})=\frac{1}{g}\cdot\sum_{a=1}^{Q} \sum_{b=a+b_{\min}+1}^{Q} \Theta\left(\varepsilon-\left\|\Psi(t_{a})-\Psi(t_{b})\right\|\right)$. (S9)

$g=\frac{\left(Q-b_{\min}\right)\left(Q-b_{\min}-1\right)}{2}$ is the total number of vector pairs within a task-epoch set *q*, *b_min_* is a temporal window to be defined below, Θ is the Heaviside function, and ε is a radius of a sphere centered on the current low-dimensional vector **Ψ**(*t_a_*). Therefore, the correlation sum *S* is the probability that trajectory points **Ψ**(*t_b_*) which are ahead of the reference point **Ψ**(*t_a_*) revisit its spatial neighborhood ε. The correlation dimension measures how this quantity scales with ε [29]. Typically, for a reliable estimation, only ε-neighbourhoods containing >10^3^ vectors were considered [29], hence correlation dimensions cannot be in principle estimated in very sparse high-dimensional spaces as earlier indicated.

The minimum time-span between samples (*Δsamples*), termed *b_min_* here, assures that only genuinely geometrical properties of the putative attractors are assessed and not relations simply induced by short-term autocorrelations along different trajectories [85]. To ensure this, *S*(ε,*Q*) (i.e. *S*(ε,*Q,b_min_*) ) must be invariant with regards to ε for sufficiently large *b_min_*, i.e. *b_min_* was chosen such that *S*(ε,*Q,b_min_*) values were nearly constant for a large ε -range. Figure S4 shows that *S*(ε,*Q*) during the Correct Choice Training epoch (TrC) fluctuates considerably across the ε -range for *Δsamples*<30, but for *Δsamples*>30 its variation is less than 5% across the whole ε-range evaluated [29, 37]. This is also valid for all other task-epochs, thus *b_min_* was conservatively fixed at 30 time bins.

Thus, by examining how *S*(ε,*Q,b_min_*) scales with ε, a dimension for the putative task-epoch attracting set can be derived. Here we used the Takens maximum likelihood estimator [38, 84] computed from the longest linear plateau as shown in Figure S4, i.e. the *scaling* region where *d_2_* is constant across some range (with a linear fit *R*^2^ >0.95±0.01). The value ε_0_ = ε*_t_*- ε_t-1_= σ(/**Ψ**/) 10^-3^ where ε_0_ is the minimum value of ε considered and σ(/**Ψ**/) is the maximum one. Since due to the short duration of the reward periods these criteria were not met for these task epochs and *d_2_* was not computed for these epochs**.** We also computed the slope of the curve *ln S*(ε,*Q,b_min_*) -*ln* ε within the ε-region as defined above, which gave another estimate of the correlation dimension very close to the Takens estimator, $\bar{d}_{2}^{slope}=1.03\pm0.12$. As shown in Figure S4, the estimates for *d_2_* were all close to 1. This suggests that three-dimensional maximum variance axes should preserve the attracting properties of high-dimensional trajectories. This is consistent with recent studies on kernel-PCA which suggest that typically few of the kernel-PCA components define a low-dimensional subspace which contains most of the relevant information [76].
